# MicroRNA-Containing T-Regulatory-Cell-Derived Exosomes Suppress Pathogenic T Helper 1 Cells

**DOI:** 10.1016/j.immuni.2014.08.008

**Published:** 2014-09-18

**Authors:** Isobel S. Okoye, Stephanie M. Coomes, Victoria S. Pelly, Stephanie Czieso, Venizelos Papayannopoulos, Tanya Tolmachova, Miguel C. Seabra, Mark S. Wilson

(Immunity *41*, 89–103, July 17, 2014)

## Main Text

In the version of this paper originally published online, the accession number for the microarray data presented in the paper was omitted. This omission has now been corrected, and the journal regrets the error.

