# Supplementary material for: MicroRNA-Containing T-Regulatory-Cell-Derived Exosomes Suppress Pathogenic T Helper 1 Cells
Source: Immunity. 2014 Jul 17;41(1):89–103. doi: 10.1016/j.immuni.2014.05.019 (PMC4104030; doi:10.1016/j.immuni.2014.05.019)
Supplement: Document S1. Figures S1–S7 and Supplemental Experimental Procedures [file mmc1.pdf]

Immunity, Volume *41*

Supplemental Information

microRNA-Containing T-Regulatory-Cell-Derived Exosomes Suppress Pathogenic T Helper 1 Cells

Isobel S. Okoye, Stephanie M. Coomes, Victoria S. Pelly, Stephanie Czieso, Venizelos Papayannopoulos, Tanya Tolmachova, Miguel C. Seabra, and Mark S. Wilson

Supplementary Figure S1

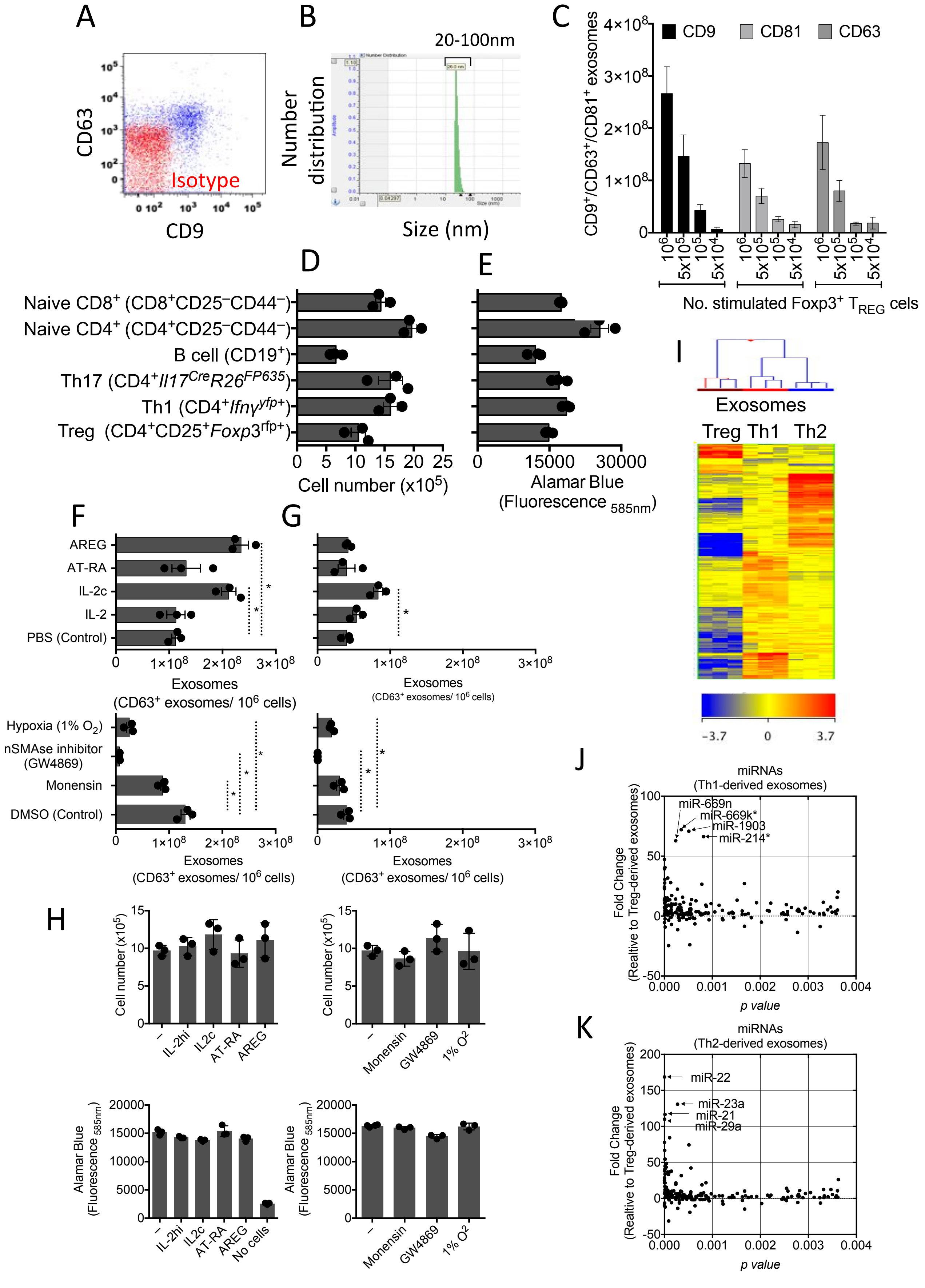

Supplementary Figure S2

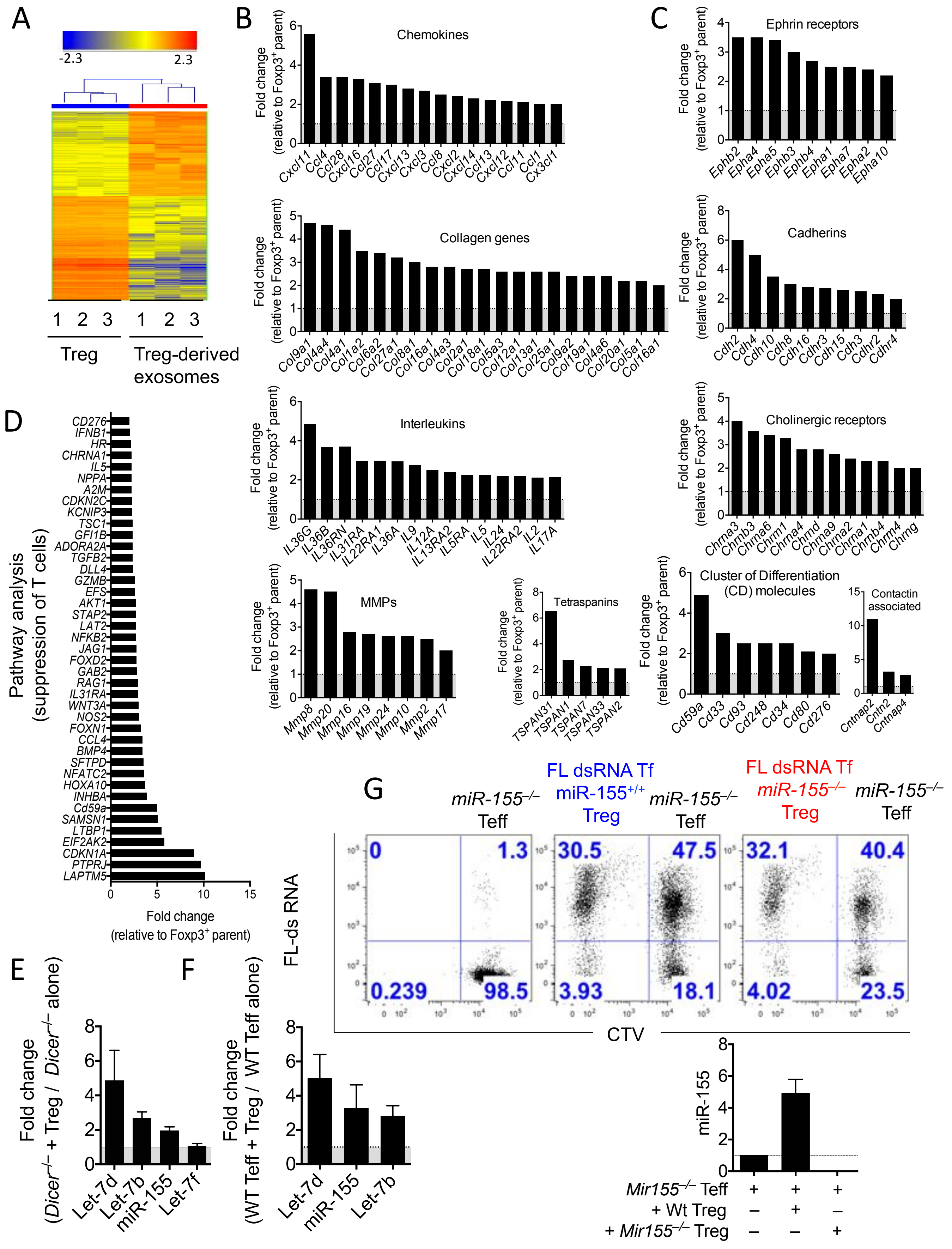

Supplementary Figure S3

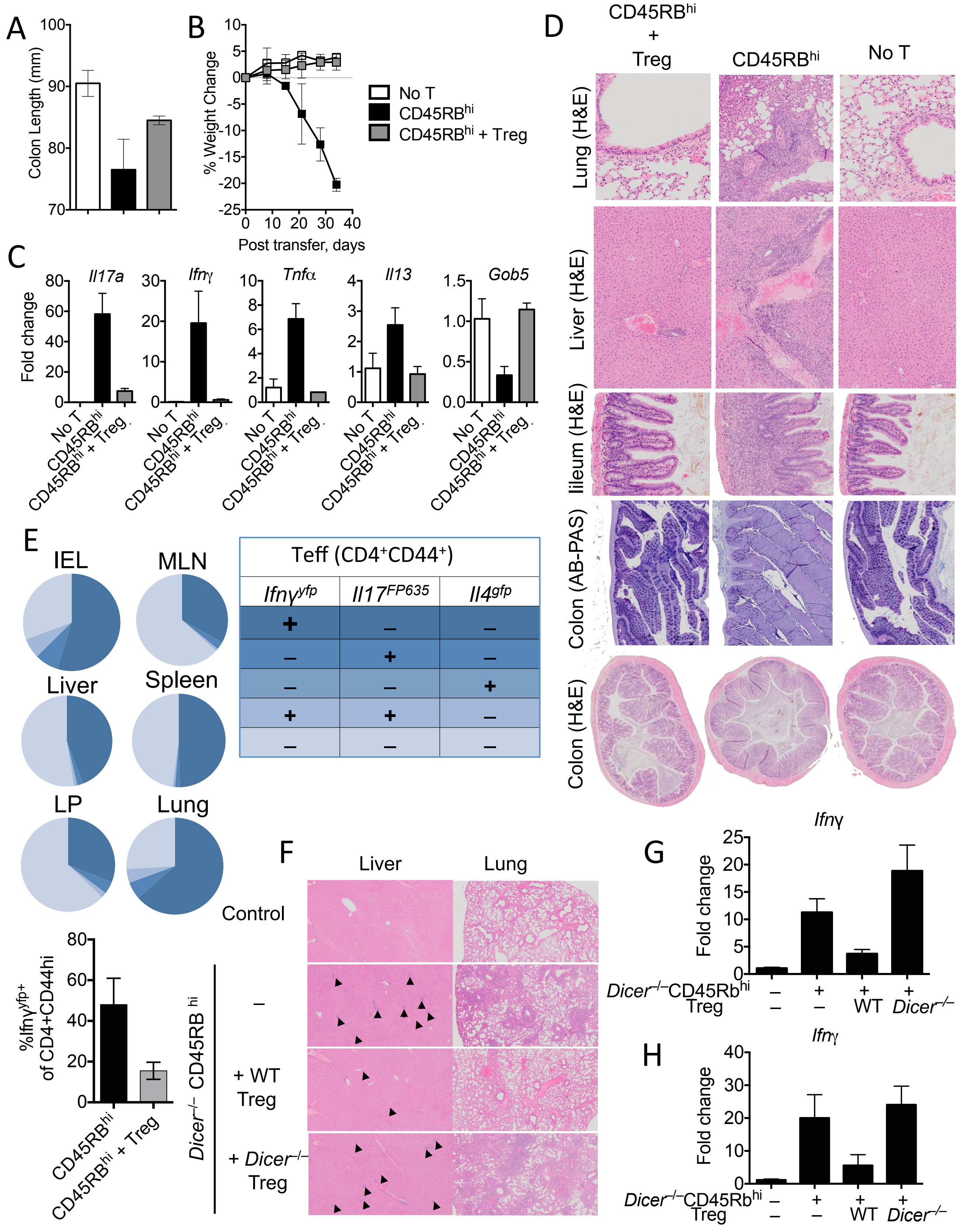

Supplementary Figure S4

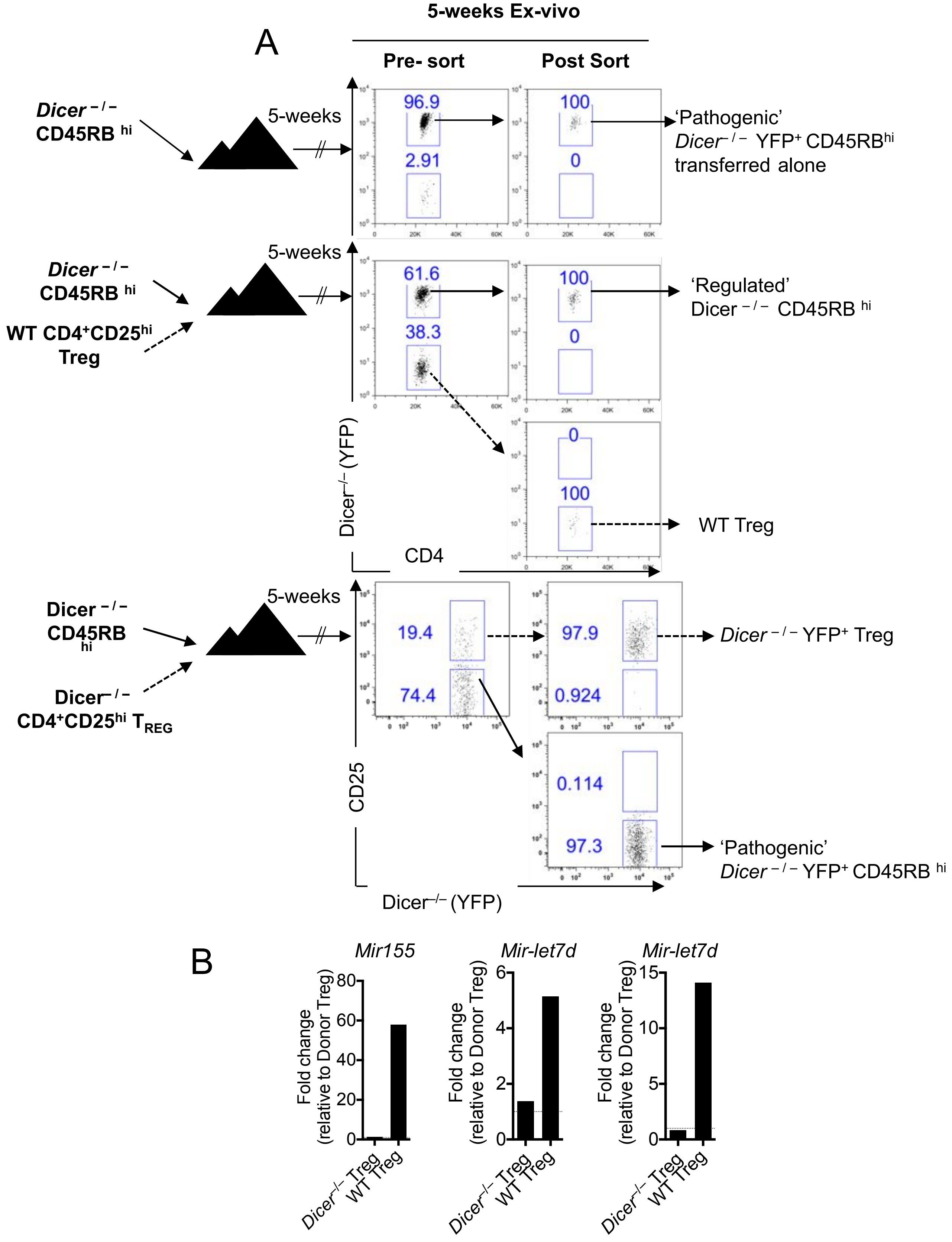

Supplementary Figure S5

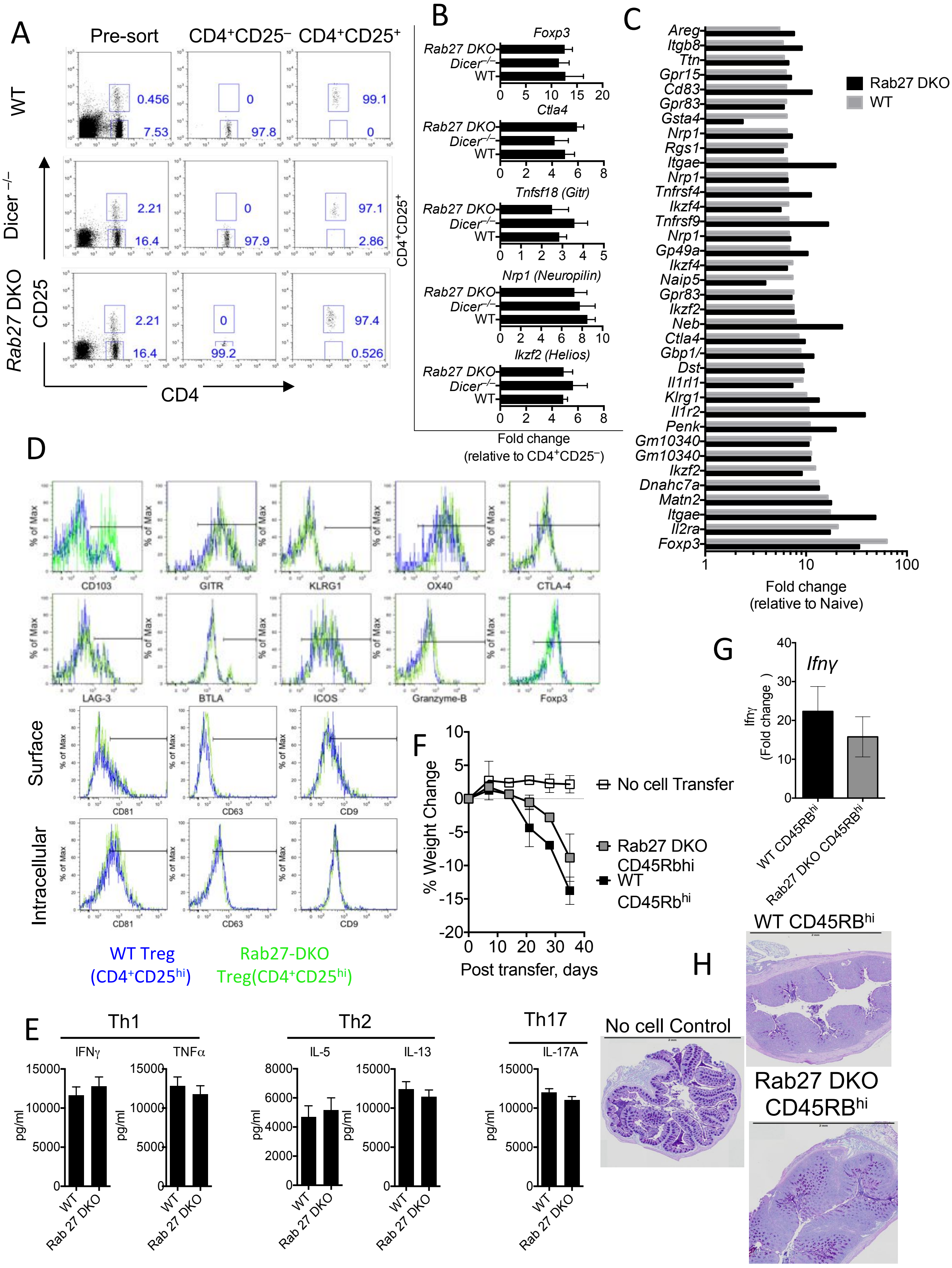

Supplementary Figure S6

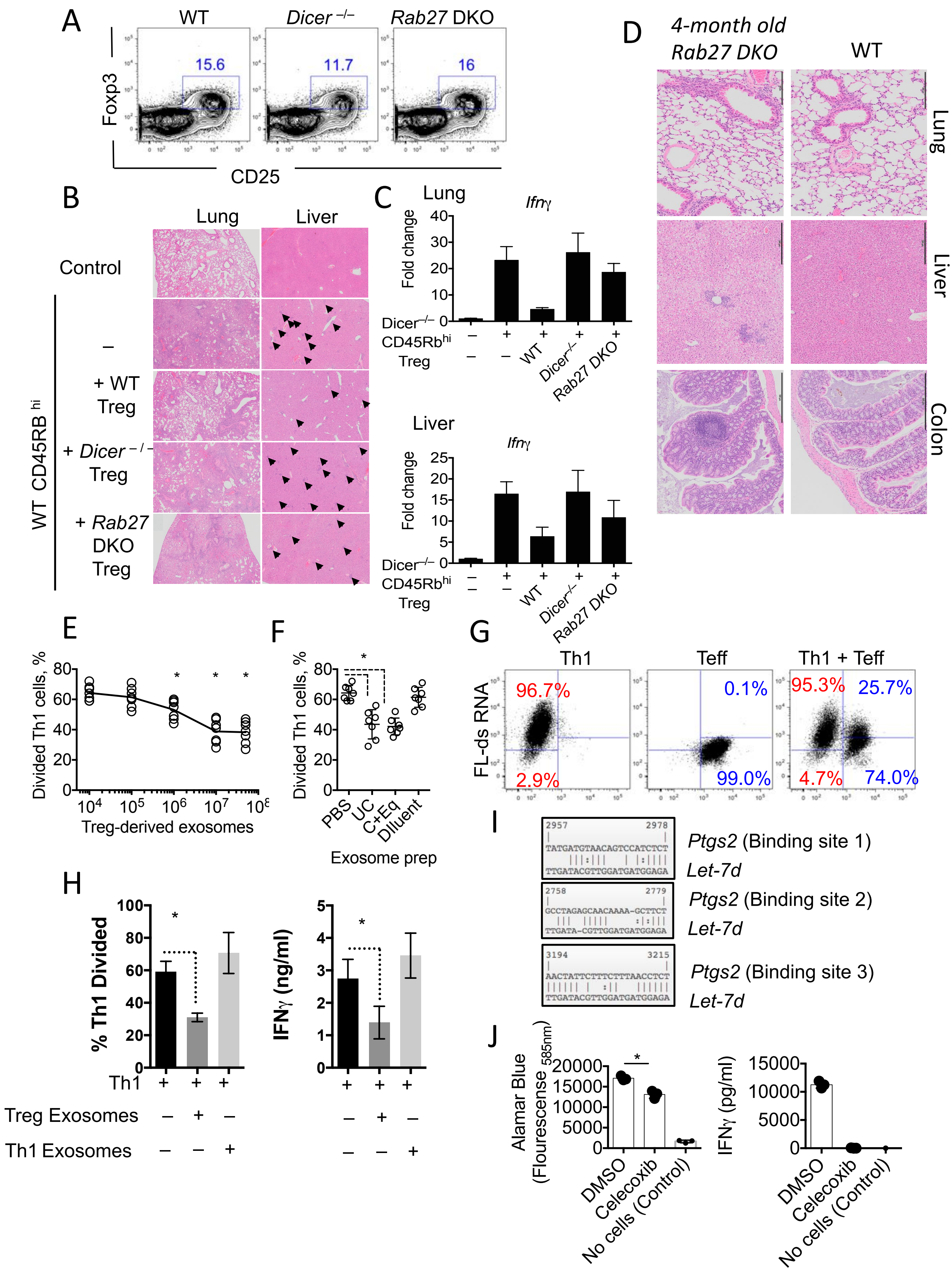

Supplementary Figure S7

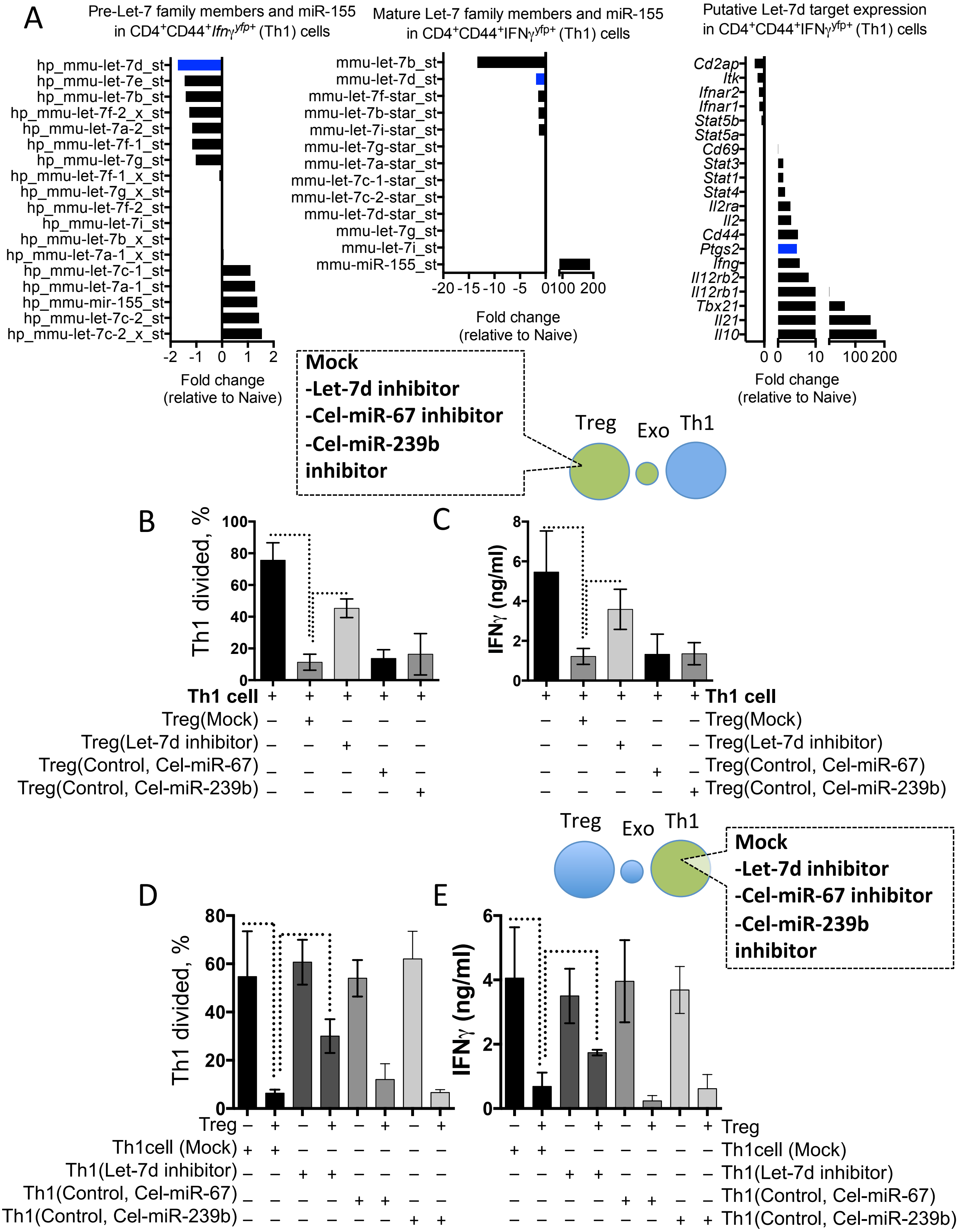

Supplementary Figures and Tables

Figure S1, related to Figure 1.

Treg- Th1 and Th2-derived exosome characteristics and cellular proliferation.

A, Treg cell derived exosomes were stained with anti-CD63 and anti-CD9 antibodies and analysed on a BD LSRII.

B, Purified exosomes were analysed using dynamic light scatter, giving a profile between 20-100nm in diameter.

C, Treg cell-derived exosomes were quantified using a CD9, CD81 or CD63 ELISA, with a known concentration of CD9, CD81 or CD63 used as a standard curve.

D-E, Following stimulation, as in Figure 1, cells were quantified by conventional trypan blue dye exclusion (left) and by Alamar blue fluorescence in culture supernatant (right).

F and G (top), Exosomes were quantified from the supernatant of Treg cells (**F**) or activated naïve cells (**G**) stimulated for 3 days with anti-CD3 (1μg/ml) and anti-CD28 (5μg/ml) with the addition of IL-2 (500ng/ml), IL-2c (5ng/ml of IL-2 in complex with 50μg/ml of anti-IL-2, JES6-1A2), AT-RA (10nM) or amphiregulin, (AREG, 100ng/ml), as indicated.

F and G, (bottom), Exosomes were quantified in the supernatant of Treg cells (**F**) or activated naïve T cells (**G**) cells cultured under normoxia (20% O_2), hypoxia (1% O_2), with a ceramide biosynthesis inhibitor (GW4869, 10μM) or monensin (10μM) for the last 6 hours of culture, as indicated. A representative of 3 experiments shown. * denotes $P<0.05$.

H, Treg cells were quantified by conventional trypan blue dye exclusion (top) and by Alamar blue fluorescence in culture supernatant (bottom) following the indicated treatments.

I, Treg cell-derived exosomes contain different miRNAs compared to Th1 or Th2 cell-derived exosomes. RNA was isolated from 3 biological replicates of Treg cell, Th1 cell or Th2 cell-derived exosomes and used for transcriptional analysis. Heat-map representing miRNA expression in Treg, Th1 cells or Th2 cells.

J, Dot plot showing miRNA expression in Th1- derived exosomes, relative to expression in Treg cell derived exosomes, with respective p value on x-axis.
K, Dot plot showing miRNA expression in Th2- derived exosomes, relative to expression in Treg cell derived exosomes, with respective p value on x-axis.

Figure S2, related to Figure 1.
mRNA content of Treg-derived exosomes and transfer of miRNAs from Treg to Teff cell.
A, RNA was isolated from 3 biological replicates of Treg cells and Treg-derived exosomes and used for transcriptional analysis. mRNA transcripts in Treg cell-derived exosomes are expressed relative to mRNA transcripts in Treg cells.
B-C, Expression of secreted (**B**) and surface (**C**) mRNA transcripts in Treg cell-derived exosomes are highlighted.
D, Ingenuity Pathways Analysis of differentially regulated mRNA transcripts in Treg-derived exosomes.
E, RNA was extracted from CD45.1$^+$*Cd4*^{Cre} *Dicer*$^{fl/fl}$ *R26*^{eyfp}CD4$^+$CD25$^-$ Teff cells after being cultured alone or with WT Treg cells, as in **Figure 3**. Expression of Let-7b, d and f and miR-155 was measured by qRT-PCR.
F, RNA was extracted from WT CD45.1$^+$CD4$^+$CD25$^-$ Teff cells after being cultured alone or with WT Treg cells, as in **Figure 3**. Expression of Let-7b, miR-155 and Let-7d was measured by qRT-PCR and expressed relative to WT Teff cells cultured alone.
G, CD45.1$^+$ miR-155-sufficient Treg cells were co-cultured with CD45.2$^+$ *miR-155*$^{-/-}$ Teff cells for 48 hours before being separated. RNA was extracted from purified CD45.2$^+$ *miR-155*$^{-/-}$ Teff cells after co-culture and miR-155 was quantified by RT-PCR. A representative of 3 experiments shown. * denotes P<0.05.

Figure S3, related to Figure 4.
Treg cell-mediated suppression of systemic inflammation.
Analysis of disease in mice following transfer of *Dicer*$^{-/-}$ CD4$^+$CD45RB^{high} T cells with or without co-transfer of WT and *Dicer*$^{-/-}$ Treg (CD4$^+$CD25^{hi}) cells.
A, Colon length measured 5 weeks post cell transfer.

B, Weekly weight measurements following cell transfer.

C, RNA expression of several inflammatory and mucous-associated genes in the colon of mice 5 weeks post cell transfer.

D, Histopathology of lung, liver, small and large intestine 5-weeks post cell transfer.

E, Percentage of cytokine^{+} cells ($Ifn\gamma^{yfp}$ $Il17a^{Cre}R26^{eFP635}$ $Il4^{gfp}$) in the intra-epithelial cells (IEL), mesenteric lymph nodes (MLN), liver, spleen, lamina propria (LP) and lung 5 weeks post cell transfer. A representative of 3 experiments shown. * denotes P<0.05.

F, Inflammatory foci in the liver and lung of mice 5-weeks post transfer of CD45RB^{hi}CD45.1^{+}$Cd4^{Cre}Dicer^{fl/fl}$ $R26^{eyfp+}$ cells alone or with WT or $Dicer^{-/-}$ Treg cells.

G and H, Expression of $Ifn\gamma$ in the liver (**G**) or lung (**H**) of mice 5-weeks post cell transfer. A representative of 3 experiments shown.

Figure S4, related to Figure 5.

Model of cell transfer and re-isolation.

A, Model and FACS plots of re-isolated ex-vivo $Dicer^{-/-}$ YFP^{+}CD4^{+}CD25^{-} cells and Treg (YFP^{-}CD4^{+}CD25^{+}) cells, 5 weeks post cell transfer from mice that received either $Dicer^{-/-}$ CD45RB^{hi} cells alone ('Pathogenic'); $Dicer^{-/-}$ CD45RB^{hi} cells ('Regulated') with WT Treg cells; $Dicer^{-/-}$ CD45RB^{hi} cells with $Dicer^{-/-}$ Treg cells. $Dicer^{-/-}$ Treg were identified as YFP^{+}CD4^{+}CD25^{+}.

B, Expression of miR-155, Let-7b and Let-7d in WT and $Dicer^{-/-}$ Treg cells, isolated 5-weeks post cell transfer, expressed relative to donor WT or $Dicer^{-/-}$ Treg cells prior to transfer. A representative of 3 experiments shown.

Figure S5, related to Figure 5.

***Rab27*-DKO T cells (Treg and Th cells) are similar to WT T cells (Treg and Th cells).**

A, Treg (CD4^{+}CD25^{+}) and naïve T cells were FACS purified from WT, $Cd4^{Cre}Dicer^{fl/fl}R26^{eyfp+}$ and *Rab27-DKO* mice.

B, Expression of Treg -associated genes determined by qRT-PCR and expressed relative to naïve CD4^{+}CD25^{-} T cells.

C, Expression of top 40 genes in WT and *Rab27-DKO* Treg cells, relative to naïve T cells.

D, Expression of surface (CD103, GITR, KLRG1, OX40, LAG-3, BTLA and ICOS) and intracellular (CTLA-4, Granzyme-B, Foxp3) Treg -associated proteins in WT (**Blue**) and *Rab27*-DKO (**Green**) Treg cells.

E, WT and *Rab27*-DKO Naïve T cells ($CD4^{+}CD44^{-}CD25^{-}$) were polarised under Th1, Th2 and Th17 conditions in vitro. Th1 (IFNγ, TNFα), T_H2 (IL-5 and IL-13) and T_H17 (IL-17A)-associated cytokines measured in cell culture supernatant after 7 days of culture.

F, Analysis of disease in *Rag2*$^{-/-}$ mice following transfer of WT or *Rab27*-DKO $CD4^{+}CD45RB^{high}$ T cells.

G, Colonic *Ifnγ* expression measured, 5-weeks post transfer.

H, Histopathology of large intestine 5-weeks post cell transfer. A representative of 2 experiments shown with 5 mice per group per experiment.

Figure S6, related to Figure 5.

***Rab27*-DKO Treg cells fail to prevent systemic T cell mediated disease. Treg-derived, but not Th1-derived, exosomes suppress Th1 cell proliferation and contain Let-7d which targets *Ptgs2* (Cox2).**

Analysis of Treg function in vivo following transfer of $CD4^{+}CD45RB^{high}$ T cells with or without transfer of $CD4^{+}CD25^{hi}$ WT, *Dicer*$^{-/-}$ or *Rab27-DKO* Treg cells.

A, Percentage of $CD25^{+}Foxp3^{+}$ cells in the CD4 compartment of the mesenteric lymph nodes of mice 5-weeks post cell transfer.

B, Inflammatory foci in the lung and liver of mice 5-weeks post transfer of WT $CD45RB^{hi}$ cells alone or with WT, *Dicer*$^{-/-}$ or *Rab27-DKO* Treg cells.

C, Expression of *Ifnγ* in the lung (left) or liver (right) of mice 5-weeks post cell transfer.

D, Inflammatory foci in the lung, liver and large intestine of 4-month old *Rab27-DKO* mice, relative to a 4-month old WT mouse**.** A representative of 2 experiments shown.

E, Naive T cells were cultured under Th1 conditions for 3 days before labeling with cell trace violet and culturing with increasing numbers of fresh Treg-derived exosomes. Cell trace violet dilution was assessed after a further 3

days of culture with exosomes and soluble anti-CD3 (1μg/ml) and anti-CD28 (5μg/ml).

F, Treg cell-derived exosomes were isolated by 2 different methods. **UC**, Treg cell supernatant from stimulated Treg cells was centrifuged at 300g for 10 minutes. The supernatant was recovered for further centrifugation at 2,000g for a further 30 minutes. The resulting supernatant was further centrifuged at 10,000g for 30 minutes. Finally, this supernatant was used to pellet exosomes at 100’000g for 2 hours. **C+Eq,** exosomes were recovered from Treg cell supernatant by a combination of centrifugation and Exoquick isolation. Treg cell supernatant from stimulated Treg cells was centrifuged at 300g for 10 minutes. The supernatant was recovered for further centrifugation at 2,000g for a further 30 minutes. The resulting supernatant was further centrifuged at 10,000g for 30 minutes. Finally, instead of 100’000g, the supernatant was mixed with Exoquick, as per manufacturers recommendation. Exosomes were re-suspended in PBS. **Diluent**, cell-free supernatant was used in the C+Eq, method, as above, as a control for the Exoquick procedure. 10^7 Treg -derived exosomes from each method were added to cell trace violet-labeled Th1 cells. Cell trace violet dilution was assessed after a further 3 days of culture with exosomes and soluble anti-CD3 (1μg/ml) and anti-CD28 (5μg/ml). A representative of 2 experiments shown. * denotes $P<0.05$.

G, Th1 cells transfer RNA to Teff cells, but are not suppressive. Th1 cells were transfected with FL-dsRNA and co-cultured with untransfected CD45.1+ Teff cells. After 24 hours, co-cultured cells were analysed by flow cytometry.

H, Exosomes were purified from Treg or Th1 cell cultures, quantified and added (10^7) to cell trace violet-labeled Th1 cells. Cells were cultured for 3 days before analysis of cell division and IFNγ measurement in supernatant.

I, Let-7d is predicted to target many genes involved in Th1 cell differentiation and effector function, including *Ptgs2***.**

J, Th1 cells were cultured with a Cox-2 inhibitor (Celecoxib, 50μM) or DMSO for 3 days. Alamar blue fluorescence and IFNγ secretion in culture supernatant were determined on day 3. A representative of 2 experiments shown. * denotes $P<0.05$.

Figure S7, related to Figure 6 and 7.

miRNAs and mRNA in Th1 cells suggest that Let-7d targets Ptgs2. Let-7d inhibitor, but not control inhibitor, treated Treg or Th1 cells, compromises Treg-mediated suppression.

A, Naïve $CD4^{+}CD25^{-}CD44^{-}IFN\gamma^{yfp-}$ cells were polarized under Th1 conditions for 1 week before FACS sorting $IFN\gamma^{yfp+}$ cells. miRNA and mRNAs were determined in FACS purified $cytokine^{+}$ Th1 $CD4^{+}IFN\gamma^{yfp+}$ cells by microarray.

B and C, Freshly isolated WT Treg cells were either MOCK transfected, transfected with Let-7d miRNA short hairpin inhibitors (100nM), or transfected with control, Cel-miR-67 or Cel-miR-239b short hairpin inhibitors (both at 100nM), as indicated. Violet cell trace-labeled Th1 cells were co-cultured at an equal ratio with treated Treg cells, as indicated. Proliferation of violet cell trace-labeled Th1 cells (**B**) and secreted IFNγ (**C**) was determined after 3 days of culture with soluble anti-CD3 (1μg/ml) and anti-CD28 (5μg/ml).

D and E, Violet cell trace-labeled Th1 cells were either MOCK transfected, transfected with Let-7d miRNA short hairpin inhibitors (100nM), or transfected with control, Cel-miR-67 or Cel-miR-239b short hairpin inhibitors (both at 100nM). Treated Th1 cells were co-cultured at an equal ratio with Treg cells, as indicated. Proliferation of violet cell trace-labeled Th1 cells (**D**) and secreted IFNγ (**E**) was determined after 3 days of culture with soluble anti-CD3 (1μg/ml) and anti-CD28 (5μg/ml). A representative of 2 experiments shown. * denotes $P<0.05$.

Supplementary Tables

Table S1, related to Figure 1.

miRNA transcripts in Treg-derived exosomes.

miRNAs identified in Treg cell-derived exosomes with fold change of miRNAs relative to miRNA expression in parental Treg cells.

Table S2, related to Figure 1.

mRNA transcripts in in Treg-derived exosomes

A list of selected mRNAs identified in Treg cell-derived exosomes with fold change of mRNA expression relative to mRNA expression in parental Treg cells.

Table S3, related to Figure 3.

Transcriptional changes in *Dicer*$^{-/-}$ T cells following co-culture with WT Treg cells.

mRNA and miRNA expression in *Dicer*$^{-/-}$ T cells after culturing with WT Treg cells, relative to *Dicer*$^{-/-}$ T cells cultured alone.

Supplementary Experimental Procedures

***Assessment of colitis and systemic inflammation*.** Mice were monitored daily and weighed weekly. Any mice showing clinical signs of severe disease were sacrificed according to the United Kingdom Animals Scientific Procedures Act of 1986. Analysis of disease was carried out 5-weeks post cell transfer. For histopathological analysis tissues were removed, fixed in 4% paraformaldehyde in PBS, embedded in paraffin and stained with Hematoxylin and eosin (H&E), Giemsa or Alcian Blue-Periodic Acid Schiff, as indicated. Cells were recovered from various tissues as indicated and re-stimulated for cytokine analysis, as below. For Let-7d inhibitor treated Treg transfer experiments, primary Treg cells were FACS-purified from WT mice and transfected with Let-7d inhibitors with 100nM of FL-dsRNA (Thermo Scientific Dharmacon). Mock or Let-7d inhibitor transfected cells were then washed extensively with 10^5 cells transferred i.v. into recipient mice on week 2, 3 and 4.

In vitro cell culture, reagents and stimulation assays. Primary cells were isolated from naive or diseased spleen, lymph nodes or tissue, as indicated, by mechanical disruption and stained with anti-mouse CD4 (RM4-5), CD3ε (17A2), CD44 (IM7), CD25 (PC61), CD45.1 (A20), CD45RB (16A), CD19 (1D3), CD8 (53-6.7) antibodies diluted in PBS with 0.1% FCS. For Treg analysis, cells were stained with CD103 (M290), GITR (DTA-1), KLRG1 (2F1), OX40 (OX-86), CTLA-4 (eBio20A), LAG-3 (eBioC9B7W), BTLA (6F7), ICOS (7E.17G9), Granzyme-B (16G6), Foxp3 (FJK-16s). For in vitro T cell polarization experiments, FACS-purified naïve T cells ($CD4^+CD44^-$ $CD62L^{hi}CD25^-$*$Ifn\gamma^{yfp-}$* or *$Il17^{Cre}R26Re^{FP635-}Il4gfp^-$*) were FACS purified and polarised under T_H1 (IL-12, 10ng/ml; anti-IL-4, 10µg/ml) or T_H17 (IL-6, 10ng/ml; TGFβ, 1ng/ml; anti-IFNγ, 10µg/ml, anti-IL-4, 10µg/ml) conditions, prior to FACS sorting of $cytokine^+$ cells. Unless otherwise stated, Th1 cells were FACS purified $CD4^+CD44^+$*$Ifn\gamma^{yfp+}$* cells. For intracellular cytokine staining, cells were stimulated with PMA (5ng/ml) and Ionomycin (500ng/ml) for 3 hours with Brefeldin A (5µg/ml) added after 1 hour. Cells were

subsequently fixed and permeabilised with BD Cytofix/cytoperm kit (BD). In some assays, primary FACS-purified Treg cells were treated with IL-2 (high dose- 500ng/ml), IL2complex (IL-2, 5ng/ml in complex with anti-IL-2, JES6-1A2, 50µg/ml), all-trans retinoic acid (AT-RA,10nM), amphiregulin (AREG, 100ng/ml), Monensin (10µM), GW4869 (10µM) or under hypoxic conditions (1% O_2) in a hypoxia workstation chamber (Ruskin Invivo$_2$ 400, Ruskin). AlamarBlue (Invitrogen) was used to monitor cellular density / proliferation inferred from the metabolic activity, according to manufacturers guidelines. For Cox-2 inhibition, Celecoxib or DMSO, was added to T_H1-polarising cultures on day 0. IFNγ and cellular proliferation was determined on day 3.

Microarray analysis. For microarray analysis, cDNA was generated from total RNA and quality was determined using an Agilent BioAnalyzer. cDNA was hybridized to Affymetrix GeneChip mouse 430.2 arrays or mouse miRNA 3.0 arrays. Hybridization, fluidics and scanning were performed according to standard Affymetrix protocols (http://www.affymetrix.com). All cel files, representing individual samples, were normalized by using the sketch RMA method within expression console (http://www.Affymetrix.com) to produce the analysed cel files. The array data were then normalized and analysed using Agilent GeneSpring software. An ANOVA was performed to obtain multiple test corrected p-values using the false discovery rate method at the 0.1 significance level and was combined with fold change values ≥2.0. The resulting data were analysed with IPA (Ingenuity® Pathway Systems, www.ingenuity.com).

miRNA mimic and hairpin inhibitor transfection. Cells were rested in exosome-free media, washed and co-cultured with T cells, B cells or dendritic cells, as indicted, for 24-48 hours. In some experiments, cells were stained with celltrace violet (5µM, Invitrogen), cultured in separate compartments separated in a transwell system with 0.4µM pore size (Corning) or cultured with both T cells and dendritic cells for 24 hours. For confocal microscopy, transfected and non-transfected cells were stained with anti-mouse CD45.1 and analysed using a Leica SP1 confocal microscope.

Exosome isolation and analysis. Throughout these studies, complete media with exosome-depleted FCS (following 100’000g centrifugation) was used (Thery et al., 2006). Exosomes were purified by a combination of ultracentrifugation and using Exoquick solution (SBI System Bioscience) (Taylor et al., 2011), as previously reported and tested (King et al., 2012). Cell supernatant was centrifuged at 300g for 10 minutes. Cell-free supernatant was further centrifuged at 2,000g and then 10,000g to remove dead cells and cell debris. Exosomes were then purified from the resulting supernatant using Exoquick solution (Taylor et al., 2011). Exosomes recovered by this method were functionally comparable to exosomes recovered by 100’000g ultracentrifugation method (**Figure S6**). Exosomes were quantified using a CD63, CD9 and CD81 ELISA, with a standard curve of known $CD63^+$ $CD9^+$ or $CD81^+$ exosomes, respectively (SBI System Bioscience). In some experiments, exosomes were stained with anti-CD63 (NVG-2) and anti-CD9 (MZ3) and analysed using a BD LSR II flow cytometer. The size distribution of purified exosomes was also confirmed using dynamic light scatter (DLS, Malvern instruments). For in vitro assays, a titration of purified exosomes from 10^4 to 5 x 10^7 was used with 10^4 T_H cells (**Figure S6**). In all other experiments, unless indicated, 10^7 T cell-derived exosomes were used.
All FACS analysis was performed using a BD LSRII and TreeStar FlowJo and all cell sorting was performed using a BD Aria II with CellQuest software.
