# Supplementary material for: MicroRNA-Containing T-Regulatory-Cell-Derived Exosomes Suppress Pathogenic T Helper 1 Cells
Source: Immunity. 2014 Jul 17;41(1):89–103. doi: 10.1016/j.immuni.2014.05.019 (PMC4104030; doi:10.1016/j.immuni.2014.05.019)
Supplement: Document S2. Article plus Supplemental Information [file mmc5.pdf]

CellPress

MicroRNA-Containing T-Regulatory-Cell-Derived Exosomes Suppress Pathogenic T Helper 1 Cells

Isobel S. Okoye,[1] Stephanie M. Coomes,[1] Victoria S. Pelly,[1] Stephanie Czieso,[1] Venizelos Papayannopoulos,[1] Tanya Tolmachova,[2] Miguel C. Seabra,[2] and Mark S. Wilson[1,*]
[1]Division of Molecular Immunology, MRC, National Institute for Medical Research, London NW7 1AA, UK
[2]Molecular Medicine Section, National Heart and Lung Institute, Imperial College London, London SW7 2AZ, UK
*Correspondence: mwilson@nimr.mrc.ac.uk
http://dx.doi.org/10.1016/j.immuni.2014.05.019
This is an open access article under the CC BY license (http://creativecommons.org/licenses/by/3.0/).

SUMMARY

Foxp3$^+$ T regulatory (Treg) cells prevent inflammatory disease but the mechanistic basis of suppression is not understood completely. Gene silencing by RNA interference can act in a cell-autonomous and non-cell-autonomous manner, providing mechanisms of intercellular regulation. Here, we demonstrate that non-cell-autonomous gene silencing, mediated by miRNA-containing exosomes, is a mechanism employed by Treg cells to suppress T-cell-mediated disease. Treg cells transferred microRNAs (miRNA) to various immune cells, including T helper 1 (Th1) cells, suppressing Th1 cell proliferation and cytokine secretion. Use of Dicer-deficient or Rab27a and Rab27b double-deficient Treg cells to disrupt miRNA biogenesis or the exosomal pathway, respectively, established a requirement for miRNAs and exosomes for Treg-cell-mediated suppression. Transcriptional analysis and miRNA inhibitor studies showed that exosome-mediated transfer of Let-7d from Treg cell to Th1 cells contributed to suppression and prevention of systemic disease. These studies reveal a mechanism of Treg-cell-mediated suppression mediated by miRNA-containing exosomes.

INTRODUCTION

Inflammation after infection, autoimmunity, or allergy is controlled to prevent immune-mediated pathologies (Nathan, 2002). T regulatory (Treg) cells prevent such inflammatory damage (Josefowicz et al., 2012; Sakaguchi, 2000; Shevach, 2000), but the precise mechanisms of suppression are incompletely understood. RNA interference (RNAi), pioneered in plants (Voinnet and Baulcombe, 1997) and worms (Fire et al., 1998), provides a mechanism of sequence-specific gene silencing, which functions in a cell-autonomous and non-cell-autonomous manner. Multivesicular bodies (MVBs) release intracellular vesicles formed from endosomal membrane, including exosomes, that have encapsulated cystolic contents (Théry, 2011). This exosomal pathway can sample miRNAs from donor cells and transfer miRNAs and other bioactive material between cells (Valadi et al., 2007), providing a vehicle for intercellular communication. Indeed, miRNA-containing exosomes have been isolated from various bodily fluids (Théry, 2011) and can regulate gene expression in various target cells (Kosaka et al., 2010).

Within the immune system, exosomes are released by a variety of innate (Théry et al., 2002; Valadi et al., 2007; Zitvogel et al., 1998) and adaptive (Bryniarski et al., 2013; Mittelbrunn et al., 2011; Raposo et al., 1996; Smyth et al., 2013) immune cells with thymus-derived exosomes capable of influencing lymphocyte differentiation (Wang et al., 2008). Furthermore, T-lymphocyte-derived exosomes can transfer miRNAs to dendritic cells, modulating gene expression in recipient cells (Mittelbrunn et al., 2011). In this study, we tested whether Treg cells release exosomes and whether miRNA-containing exosomes contribute to Treg-cell-mediated control of immune responses. In summary, we observed that Treg cells released a significant quantity of miRNA-containing exosomes and that miRNAs were transferred to Th1 cells in vitro and in vivo. Coculture and transcriptional analyses identified that Treg cells transferred Let-7d to Th1 cells, suppressing Th1 cell proliferation and IFN-γ secretion. More importantly, miRNA biogenesis and the ability of Treg cells to release exosomes were both required for Treg cells to suppress Th1 cell proliferation in vivo and prevent systemic disease.

RESULTS

Treg Cells Release Exosomes

Exosome release was quantified from different lymphocytes, including CD4$^+$ and CD8$^+$ naive T cells, T helper 1 (Th1) and Th17 cells, Treg cells, and CD19$^+$ B cells, using CD63 as an exosomal surface marker, which correlated with other exosome markers including CD9 and CD81 (Figures S1A–S1C available online). After activation of various T cells, with anti-CD3 and anti-CD28, or B cells, with CD40L and LPS, Treg cells released more CD63$^+$ exosomes per cell than other lymphocytes, despite only a minor change in cell number (Figures 1A, S1D, and S1E). Factors that regulate Treg cells, including interleukin 2 (IL-2) (Boyman et al., 2006; Fontenot et al., 2005), Amphiregulin (Areg) (Zaiss et al., 2013), or all-trans retinoic acid (AT-RA) (Nolting et al., 2009), also regulated exosome release (Figures S1F and S1G) without any appreciable change in Treg cell number or metabolic activity over the culture period (Figure S1H). Despite 60% fewer exosomes released from naive T cells compared to Treg cells, exosome release from naive CD4$^+$ T cells was also regulated by IL-2 (Figure S1G). Exosome release

CrossMark

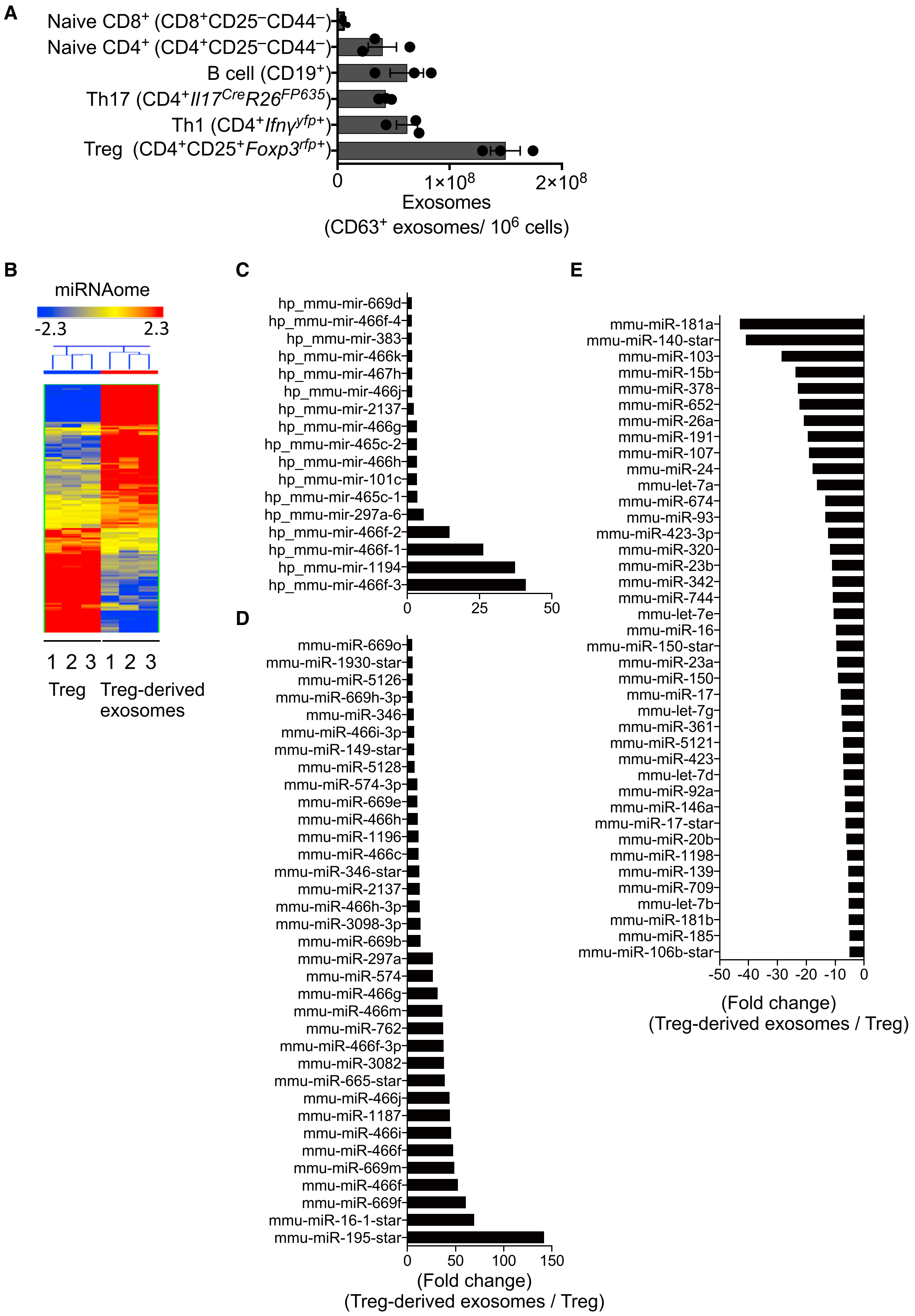

(legend on next page)

is regulated by changes in intracellular calcium (Ca2^{+}) (Savina et al., 2003), hypoxia (King et al., 2012), and sphingolipid ceramide synthesis (Trajkovic et al., 2008). Similarly, exosome release by Treg cells was regulated by the calcium ionophore, monensin, hypoxia, and ceramide biosynthesis (Figure S1F).

miRNAs are essential for Treg-cell-autonomous functions (Liston et al., 2008; Zhou et al., 2008). However, miRNAs have also been observed in exosomes, released from Jurkat T cell lines (Mittelbrunn et al., 2011) and other T cell clones (Nolte-'t Hoen et al., 2012), raising the possibility that miRNAs may also function in a non-T-cell-autonomous manner. Using genome-wide miRNA analysis, we found that Treg-cell-derived exosomes contained both premature and mature miRNAs (Figures 1B–1E, Table S1). Furthermore, the composition of miRNAs in Treg-cell-derived exosomes was distinct from that of parental Treg cells. Of note, the most abundant miRNAs in Treg-cell-derived exosomes (miR-466 family [Druz et al., 2011], miR-195 [Yang et al., 2012], and miR-16 [Cai et al., 2012]) have either proapoptotic or antiproliferative properties. Microarray analysis of Th1- and Th2-cell-derived exosomes revealed a different suite of miRNAs to those identified in Treg-cell-derived exosomes (Figures S1I–S1K), suggesting that different T cells release different miRNAs. In addition to miRNAs, genome-wide mRNA analysis identified many mRNA transcripts enriched in Treg-cell-derived exosomes (Figure S2 and Table S2). Unbiased pathway analysis of mRNA transcripts in Treg-cell-derived exosomes identified a profile consistent with the suppression of T cells (Figure S2D), suggesting that Treg-cell-derived exosomes may be involved in T cell regulation.

miRNAs Are Transferred from Treg Cells to Conventional T Effector Cells In Vitro and In Vivo

To test whther RNA was transferred between immune cells, we developed a flow-cytometry-based system using Treg cells transfected with fluorescent oligonucleotide duplexes (FL-dsRNA) (Figure 2A), allowing us to track Treg-cell-derived dsRNA in single cells. When we cocultured Treg cells with other leukocytes, Treg cells transferred FL-dsRNA to ~60% of cocultured naive T cells (CD3^{+}CD4^{+}CD25^{-}) or other Treg cells (CD3^{+}CD4^{+}*Foxp3*$^{rfp+}$CD25^{+}) and to ~40% of cocultured DCs (CD11c^{+}MHCII^{hi}) or B cells (CD3^{-}CD19^{+}) (Figure 2B). Using an in vitro suppression assay with a decreasing number of FL-dsRNA-transfected Treg cells to conventional T effector (Teff) cells, we observed a dose-dependent transfer of FL-dsRNA to Teff cells (Figure 2C), with the amount of FL-dsRNA transferred correlated with the decrease in target cell proliferation. These data suggest that transfer of RNA from Treg cells to conventional T cells may be involved in the suppression of conventional T cells.

RNA can be transferred from T cells to antigen-presenting cells (APCs) (Mittelbrunn et al., 2011) and between various APC populations (Montecalvo et al., 2012). However, addition of APCs to Treg cell and conventional T cell cocultures had no impact on RNA transfer to conventional T cells (Figure 2D). HIV-1 virus can be transferred between T cells in nanotubules (Sowinski et al., 2008) in a cell-contact-dependent manner. However, when Treg cells and conventional T cells were cultured in a transwell system, physically separating Treg cell and conventional T cells beyond the reach of nanotubes, RNA transfer was only slightly reduced (Figure 2E), suggesting transfer of RNA by an extracellular microvesicle-related mechanism.

To determine whether FL-dsRNA was being transferred into the cytoplasm of recipient conventional T cells from donor Treg cells, rather than simply decorating the surface of recipient cells, we cocultured Treg cells and conventional T cells and then FACS purified each population after coculture. Using anti-CD45.1 membrane stain on the recipient cell, we verified that the FL-dsRNA was indeed delivered inside the recipient cell (Figure 2F).

Next, we took advantage of the dependency on Dicer for mature miRNA processing (Hutvágner et al., 2001) to formally test whether miRNAs were transferred from Treg cells to conventional T cells in vitro. Treg cells from miRNA-sufficient CD45.2 WT mice were cocultured with miRNA-deficient conventional T cells from congenic CD45.1^{+}*Cd4*^{Cre}*Dicer*$^{fl/fl}$*R26*^{eyfp} (*Dicer*$^{-/-}$) mice, which were labeled with cell trace violet (Figure 3A). This system allowed us to (1) accurately discriminate and separate each population, (2) assess proliferation of *Dicer*$^{-/-}$ conventional T cells, and (3) repurify *Dicer*$^{-/-}$ conventional T cells after coculture to identify the presence of any acquired miRNAs. As expected, Treg cells suppressed (Figure 3B) and transcriptionally altered (Figure 3C) *Dicer*$^{-/-}$ T cells. Several inflammatory cytokines (*Il22*, *Il2*, *il17a*, *Ifng*) were downregulated in "suppressed" *Dicer*$^{-/-}$ T cells, with the concomitant increase in several regulatory genes (*Cd73, Foxp3, Ikzf2* [encoding Helios], and *Cd103*) (Figure 3C, Table S3), relative to *Dicer*$^{-/-}$ T cells cultured alone. Concurrently, *Dicer*$^{-/-}$ conventional T cells acquired three mature (miR-155, Let-7b, and Let-7d) and one pre-miRNA (Hp_miR-344d-2) from Treg cells (Figures 3D and S2E). Using a Dicer-sufficient (WT) congenic system with CD45.2^{+} WT Treg cells and CD45.1^{+} WT conventional T cells, we also observed an increase in miR-155, Let-7d, and Let-7b in Dicer-sufficient WT conventional T cells, when cocultured with WT Treg cells (Figure S2F), further supporting the observation that miRNAs were transferred between cells. Finally, using CD45.2^{+} *mir155*$^{-/-}$ conventional T cells as recipient cells, cocultured with CD45.1^{+} *mir155*$^{+/+}$ (WT) Treg cells, we confirmed the transfer of miR-155 from Treg to *mir155*$^{-/-}$ conventional T cells (Figure S2G). Taken together these data support the notion that Treg cells transfer specific miRNAs to conventional T cells, suppressing effector gene expression and proliferation.

The adoptive transfer of Treg-cell-depleted CD4^{+}CD45RB^{hi} T cells into T-cell-deficient mice leads to systemic inflammation (Powrie et al., 1994), which can be prevented by the cotransfer of Treg cells (Figures S3A–S3E). Despite the loss of miRNAs,

Figure 1. Treg Cells Produce More Exosomes than Other Lymphocytes that Carry Distinct miRNAs
(A) Exosomes were purified and quantified by CD63 ELISA from the supernatant of 10^{6} stimulated primary lymphocytes.
(B) Total RNA was isolated from three biological replicates of Treg cells or purified Treg-cell-derived exosomes and used for miRNA microarrays.
(C–E) Premature (C) and mature upregulated (D) and mature downregulated (E) miRNAs in Treg-cell-derived exosomes are expressed relative to expression of premature and mature miRNAs in parental Treg cells. A representative of two or three experiments shown.
Error bars are SD.

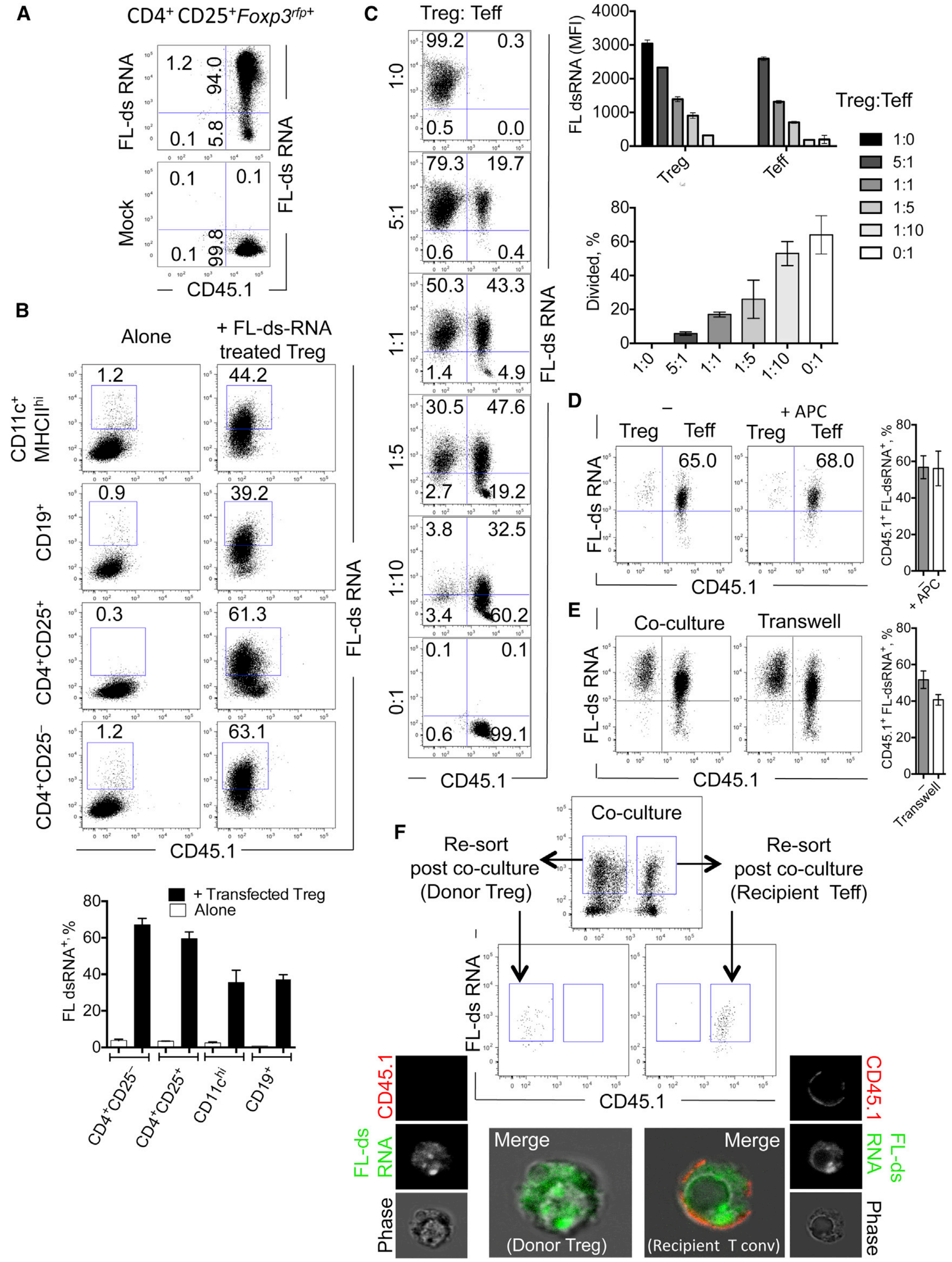

Figure 2. Treg Cells Transfer RNA to Various Leukocyte Populations In Vitro

(A) Primary Treg cells (CD4$^+$CD25$^+$*Foxp3*$^{rfp+}$) were transfected with fluorescently labeled double stranded RNA (100 nM, FL-dsRNA), rested overnight, washed, and analyzed for transfection efficiency.

(B) Splenic dendritic cells (CD11c$^+$MHCII$^+$), B cells (CD19$^+$CD4$^-$CD8$^-$CD25$^-$CD44$^-$), Treg cells (CD4$^+$CD25$^+$), and naive T cells (CD4$^+$CD25$^-$CD44$^-$) were cultured alone (left) or with an equal number of FL-ds RNA-transfected Treg cells (right). The percentage of FL-ds RNA$^+$ nontransfected cocultured cells were quantified after 48 hr of coculture.

(legend continued on next page)

Dicer$^{-/-}$ CD45RB^{hi} T cells also caused a similar wasting disease with systemic inflammation (Figures 4A and 4B) and IFN-γ production (Figures 4C, 4D, and S3F–S3H), which could be prevented by cotransfer of WT, but not *Dicer*$^{-/-}$, Treg cells. Because *Dicer*$^{-/-}$ CD45RB^{hi} cells retained pathogenicity and sensitivity to Treg-cell-mediated control, we were able to test whether miRNAs were transferred to *Dicer*$^{-/-}$ CD45RB^{hi} cells in vivo. After 5 weeks, "pathogenic" CD4$^+$*Dicer*$^{-/-}$ YFP$^+$ (*Dicer*$^{-/-}$ CD45RB^{hi} cells transferred alone) or "regulated" CD4$^+$*Dicer*$^{-/-}$ YFP$^+$ (*Dicer*$^{-/-}$ CD45RB^{hi} cells cotransferred with WT Treg cells) were recovered ex vivo to determine whether *Dicer*$^{-/-}$ cells acquired miRNAs in vivo (Figure S4A). Consistent with a suppressed state, regulated CD4$^+$*Dicer*$^{-/-}$ YFP$^+$ cells had reduced *Ifng* and *Tnf* expression (Figure 4D), compared to pathogenic CD4$^+$*Dicer*$^{-/-}$ YFP$^+$ cells. miRNA analysis of CD4$^+$*Dicer*$^{-/-}$ CD45RB^{hi} cells pretransfer and pathogenic and regulated CD4$^+$*Dicer*$^{-/-}$ YFP$^+$ cells isolated ex vivo confirmed our in vitro observations (Figure 3) and identified the presence of miR-155, Let-7b, and Let-7d in regulated CD4$^+$*Dicer*$^{-/-}$ YFP$^+$ cells, when WT Treg cells had been cotransferred (Figure 4E). In contrast, miR-155, Let-7b, and Let-7d weres not observed in pathogenic CD4$^+$*Dicer*$^{-/-}$ YFP$^+$ cells, when no Treg cells were transferred, suggesting that WT Treg cells either supported or directly transferred miRNAs to *Dicer*$^{-/-}$ cells. Relative to a housekeeping small RNA, RNU6B, regulated CD4$^+$*Dicer*$^{-/-}$ YFP$^+$ cells had almost as much miR-155, Let-7b, and Let-7d as WT Treg cells pretransfer, suggesting that a large amount of RNA was being transferred. Of note, WT Treg cells recovered ex vivo had elevated expression of miR-155, Let-7b, and Let-7d compared to WT Treg cells pretransfer (Figures 4E and S4B), suggesting that activated Treg cells also increase transcription of these miRNAs.

Within miRNA-sufficient *Rag2*$^{-/-}$ hosts, it was conceivable that the regulated CD4$^+$*Dicer*$^{-/-}$ YFP$^+$ cells acquired miRNAs from non-Treg cells. We therefore used an additional control of *Dicer*$^{-/-}$ Treg cells, cotransferred with *Dicer*$^{-/-}$ CD45RB^{hi} cells. *Dicer*$^{-/-}$ Treg cells failed to suppress disease. Furthermore, *Dicer*$^{-/-}$CD4$^+$YFP$^+$ cells from mice that were cotransferred with *Dicer*$^{-/-}$ Treg cells did not have measurable miR-155, Let-7b, or Let-7d (Figure 4E). These data demonstrate that Treg-cell-mediated suppression is accompanied by the transfer of these three, and possibly other, miRNAs from Treg cells.

Treg-Cell-Mediated Suppression Is Rab27 Dependent

Exosome release requires Rab27a and Rab27b for docking multivesicular endosomes (MVE) to Rab27 effectors on the plasma membrane (Fukuda, 2013; Ostrowski et al., 2010; Singh et al., 2013). To test the role of Rab27 and exosome release, we purified Treg cells from *Rab27*$^{ashen/ashen}$*Rab27b*$^{-/-}$ double knockout mice (Rab27-DKO) and stimulated these cells, as above. Compared to WT and *Dicer*$^{-/-}$ Treg cells, *Rab27*-DKO Treg cells failed to release exosomes with no change in cell viability (Figures 5A and 5B), confirming the requirement of Rab27 for exosome release from Treg cells. Under hypoxic conditions, exosome release was significantly curtailed in WT and *Dicer*$^{-/-}$ Treg cells (Figure 5B). Rab27-DKO Treg cells also failed to transfer FL-dsRNA from Treg cells to conventional Teff cells (Figure 5C), indicating that a Rab27-regulated exosomal pathway was responsible for transferring RNA between T cells. Furthermore, when cocultured with Th1 cells, Rab27-DKO Treg cells failed to suppress Th1 cells, similar to *Dicer*$^{-/-}$ Treg cells (Figure 5D; Liston et al., 2008; Muljo et al., 2005). These data demonstrate that Rab27 is essential for (1) exosome release from Treg cells, (2) RNA transfer from Treg cells to other T cells, and (3) proficient Treg-cell-mediated suppression in vitro.

Beyond a failure to release exosomes, purified splenic *Rab27*-DKO Treg cells were very similar to WT Treg cells, with a comparable frequency (Figure S5A), transcriptional profile (Figures S5B, qRT-PCR, and S5C, microarray), and intracellular and surface Treg cell marker and tetraspanin expression profile (Figure S5D), indicating that Rab27 was not required for Treg cell development or many other features of Treg cells. Furthermore, the deletion of Rab27 did not impact the ability of *Rab27*-DKO Th cells to secrete cytokines (Figure S5E), migrate, and mediate disease when transferred into *Rag2*$^{-/-}$ hosts in vivo (Figures S5F–S5H), indicating that Rab27a and Rab27b, unlike SNAREs and Rho GTPases (Alonso and Millán, 2001), are primarily required for the exosomal pathway.

To test whether Rab27-dependent exosome release contributed to Treg cell function in vivo, we transferred WT or *Rab27*-DKO Treg cells with WT CD45RB^{hi} T cells into *Rag2*$^{-/-}$ hosts. Similar to miRNA-ablated *Dicer*$^{-/-}$ Treg cells, *Rab27*-DKO Treg cells failed to prevent disease with significantly elevated *Ifng* expression (Figure 5E), colon shortening (Figure 5F), and weight loss (Figure 5G) with IFN-γ^+ T cell recruitment (Figure 5H), despite a similar frequency of Treg cells observed (Figure S6A). Failure of *Rab27*-DKO Treg cells to control effector T cells also led to significant colonic and systemic inflammation and *Ifng* expression (Figures 5I, S6B, and S6C), demonstrating that Rab27-dependent exosome release was essential for Treg cell function in vitro and in vivo.

Using *Dicer*$^{-/-}$ CD4$^+$CD45RB^{hi} effector cells cotransferred with WT, *Dicer*$^{-/-}$, or *Rab27*-DKO Treg cells, we identified increased amounts of miR-155, Let-7b, and Let-7d in ex vivo regulated CD4$^+$*Dicer*$^{-/-}$ YFP$^+$ cells that were cotransferred with WT, but not *Dicer*$^{-/-}$ or *Rab27*-DKO, Treg cells (Figure 5J). These data support and extend our in vitro and in vivo observations,

(C) FL-dsRNA-transfected Treg cells were cocultured with naive conventional T cells (CD4$^+$CD25$^-$CD44$^-$) at the indicated ratios on anti-CD3 (1 μg/ml)- and anti-CD28 (5 μg/ml)-coated plates for 3 days. The proliferation of conventional T cells and MFI of either transfected Treg cells and nontransfected conventional T cells were quantified by FACS.

(D) FL-dsRNA-transfected Treg cells were cocultured with naive conventional T cells (CD4$^+$CD25$^-$CD44$^-$) in the presence or absence of APCs (CD11c$^+$MHCII$^+$), as indicated, for 24 hr.

(E) FL-dsRNA-transfected Treg cells were cultured with naive conventional T cells (CD4$^+$CD25$^-$CD44$^-$) separated by a 0.4 μm filter in a transwell assay system.

(F) CD4$^+$CD25^{hi}CD45.2$^+$ Treg cells were transfected with FL-dsRNA and cocultured with CD4$^+$CD25$^-$CD44$^-$CD45.1$^+$ conventional T cells for 24 hr. After 24 hr of coculture, FL-dsRNA$^+$ Treg cells or conventional T cells were resorted and analyzed by confocal microscopy to identify the cellular location of the FL-dsRNA$^+$. A representative of three experiments shown.

Error bars are SD.

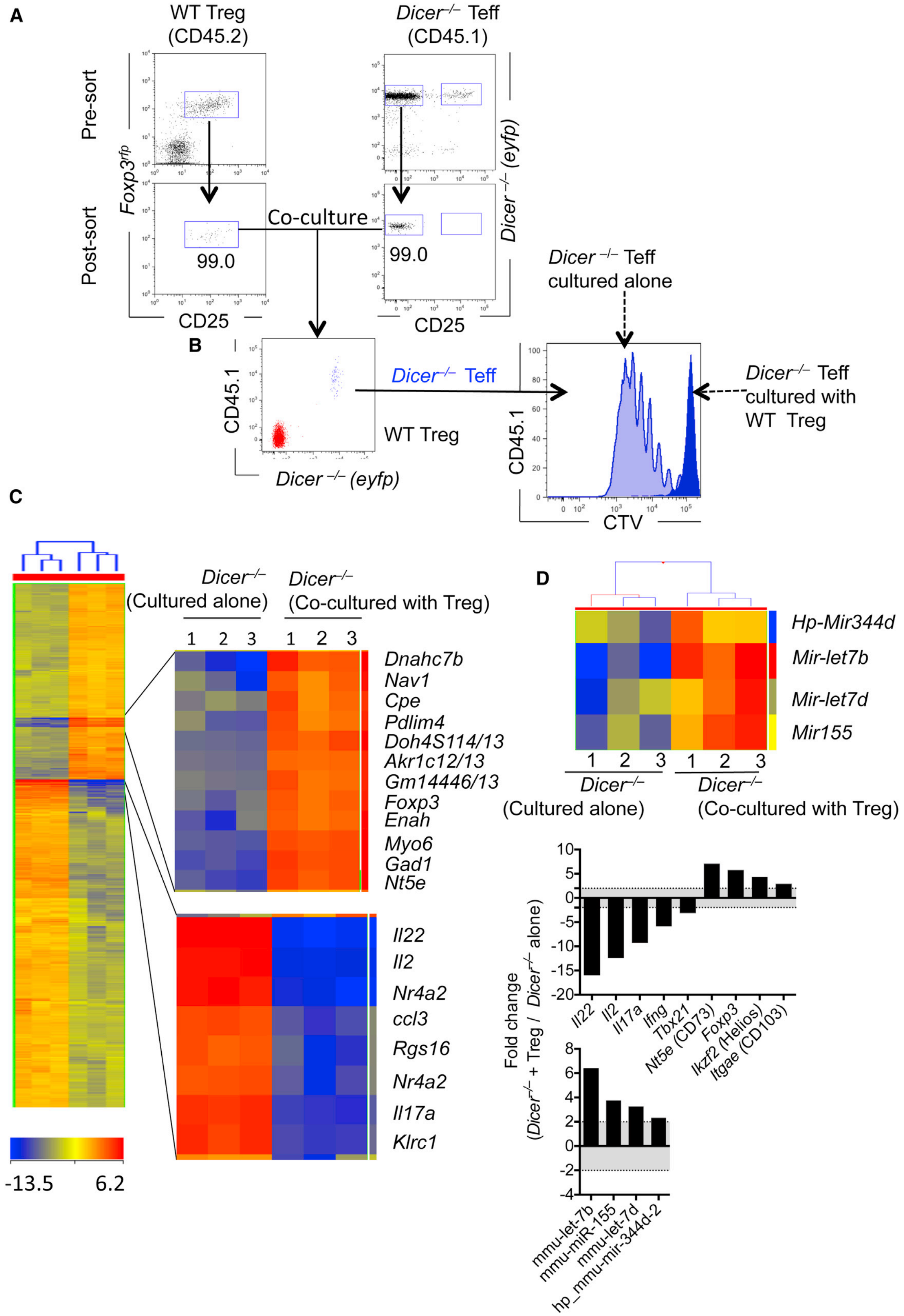

(legend on next page)

indicating that both Dicer and Rab27 sufficiency in Treg cells is required for the transfer of miRNAs, suppression of effector T cells, and prevention of disease. Of note, *Rab27*-DKO mice suffered from mild pulmonary inflammation with increased granulocyte influx and abnormal airway epithelium (Bolasco et al., 2011). Upon further examination, aged *Rab27*-DKO mice had inflammatory foci in the lung, liver, and colon, which may be due to compromised Treg-cell-mediated immune regulation (Figure S6D).

Let-7d in Treg-Cell-Derived Exosomes Contributes to Suppression of Th1 Cells

To determine whether miRNAs in Treg-cell-derived exosomes were suppressive, we purified exosomes released from WT and *Dicer*$^{-/-}$ Treg cells. WT, but not *Dicer*$^{-/-}$, Treg cells suppressed Th1 cell proliferation, as previously reported (Liston et al., 2008; Muljo et al., 2005). Similarly, 10^7 exosomes from WT, but not *Dicer*$^{-/-}$, Treg cells suppressed Th1 cell proliferation (Figures 6A, 6B, S6E, and S6F) and IFN-γ secretion (Figure 6C), indicating that exosomes were suppressive in a Dicer-dependent manner. Although Th1 cells could transfer RNA to conventional T cells in vitro (Figure S6G), Th1-cell-derived exosomes had no impact on Th1 cell differentiation (Figure S6H). Th1 cells had reduced amounts of Let-7d, compared to naive T cells (Figure S7A), and Th1-cell-derived exosomes were devoid of Let-7d (Figure S1I), providing one explanation of why Th1-cell-derived exosomes could not suppress Th1 cells.

Following the observation that miR-155, Let-7b, and Let-7d were transferred from Treg cells to conventional T cells, we tested whether miR-155, Let-7b, or Let-7d, alone or in combination, were responsible for suppression of Th1 cells. Transfection of Th1-cell-polarized WT or *Dicer*$^{-/-}$ T cells with miRNA mimics of miR-155, Let-7b, Let-7d, or all three miRNAs increased the expression of each respective miRNA (Figure 6D). Consistent with previous reports, miR-155 enhanced IFN-γ (Banerjee et al., 2010) and TNF-α secretion, without any appreciable change in proliferation (Figures 6E and 6F), indicating that miR-155 promoted, rather than inhibited, Th1 cell responses. Transfection of Th1 cells with Let-7b had little impact on IFN-γ production or proliferation (Figures 6E and 6F). In contrast, transfection with Let-7d, alone or in combination with either Let-7b or miR-155, significantly reduced *Tnf* and *Ifng* mRNA (Figure 6E), Th1 cell proliferation, and IFN-γ secretion (Figure 6F), suggesting that the transfer of Let-7d from Treg cells to Th1 cells may be an important intermediary of Treg-cell-mediated suppression of Th1 cells.

Target prediction analyses (Miranda et al., 2006) identified many putative Let-7d targets involved in Th1 cells (*Il12r*, *Tbx21*, *Stat1* and *Stat4*, and *Ifng*). We therefore analyzed the miRNA and mRNA profile of FACS-purified IFN-γ^{yfp+} Th1 cells, compared to naive T cells, and identified reduced premature and mature Let-7d with an increase in many Let-7d target genes (Figure S7A). Specifically, Let-7d was predicted to target *Ptgs2* (Cox-2), which was elevated in Th1 cells (Figures S6 and S7). Testing the requirement of Cox-2 using a selective Cox-2 inhibitor (celecoxib), we noted that Cox-2 inhibition ablated IFN-γ secretion (Figure S6J), indicating that Cox-2 is required for Th1 cell responses. Thus, Let-7d may target Cox-2 in Th1 cells to regulate IFN-γ secretion.

To test whether Let-7d from Treg-cell-derived exosomes contributed to the suppression of Th1 cell responses in vitro, we transfected Th1 cells with Let-7d hairpin inhibitors, to inhibit endogenous or exogenous Let-7d, and cocultured these cells with Treg cells or exosomes purified from Treg cells. Let-7d inhibitor treatment alone did not impact Th1 cell proliferation or IFN-γ secretion in the absence of Treg cells (Figures 7A, 7B, and S7E). The addition of Treg cells or Treg-cell-derived exosomes suppressed Th1 cell proliferation (Figures 7A and 7B) and IFN-γ secretion (Figure 7C). However, Treg cells and Treg-cell-derived exosomes failed to suppress Let-7d inhibitor-treated Th1 cells, indicating that sequestering endogenous or exogenous Let-7d in Th1 cells prevented Treg cell and Treg cell exosome-mediated suppression (Figures 7A–7C). To further test whether Let-7d from Treg cells contributed to Treg cell- and Treg-cell-derived exosome-mediated suppression, we transfected Treg cells with Let-7d hairpin inhibitors or control inhibitors (Figures 7D and S7). Let-7d inhibitor treatment reduced Let-7d by ~50% in Treg cells and by ~75% in Treg-cell-derived exosomes without any appreciable impact on cell viability (Figure 7D). Let-7d inhibitor-treated Treg cells, but not control inhibitor-treated Treg cells (Figure S7), were compromised in their ability to suppress Th1 cells, and Let-7d-depleted exosomes had completely lost their ability to suppress Th1 cells (Figures 7D and 7E). Although it remains possible that Let-7d inhibitors were transferred with Let-7d to Th1 cells in this experimental setup, taken together with the reduced Let-7d in Th1 cells (Figure S7) and the fact that Let-7d inhibitor-treated Th1 cells when cultured alone proliferated and secreted IFN-γ similar to control Th1 cells (Figures 7A and 7B, black bars), these data indicate that Let-7d contributes to Treg-cell-mediated suppression and more specifically, that Let-7d was responsible for Treg-cell-derived exosome-mediated suppression.

Finally, we tested whether Let-7d was required for Treg-cell-mediated suppression in vivo by transferring mock or Let-7d inhibitor-treated WT Treg cells into *Rag2*$^{-/-}$ hosts with WT CD45RB^{hi} cells. Transfected Treg cells survive only 7–10 days in vivo (Kelada et al., 2013), so we transferred Treg cells weekly for 3 weeks (weeks 2, 3, and 4) before analysis at week 5. Mock-transfected Treg cells prevented weight loss, *Ifng* responses, and intestinal inflammation (Figures 7F–7H). In contrast, Let-7d

Figure 3. Treg Cells Transfer miR-155, Let-7b, and Let-7d to T Cells In Vitro

(A) FACS-purified *Dicer*$^{-/-}$ cell-trace violet (CTV)-labeled conventional T cells (CD45.1$^+$*Cd4*^{Cre}*Dicer*$^{fl/fl}$*R26*$^{eyfp+}$CD4$^+$CD25$^-$) were cultured alone or with primary Treg cells (CD45.2$^+$CD4$^+$CD25$^+$*Foxp3*^{rfp}) for 3 days on anti-CD3 (1 μg/ml)- and anti-CD28 (5 μg/ml)-coated plates.

(B) Proliferation of *Dicer*$^{-/-}$ conventional Teff cells was assessed and FACS sorted.

(C and D) RNA was extracted from three biological replicates of CD45.1$^+$*Cd4*^{Cre} *Dicer*$^{fl/fl}$ *R26*^{eyfp}CD4$^+$CD25 Teff cells after being cultured alone or with WT Treg cells. Heatmaps of differentially expressed genes used for mRNA (C) or miRNA (D) analysis. Graph on right highlights expression of inflammatory and regulatory genes in CD45.1$^+$*Dicer*$^{-/-}$ conventional T cells cocultured with WT Treg cells, expressed relative to CD45.1$^+$*Dicer*$^{-/-}$ conventional T cells cultured alone. A representative of three experiments shown, with three biological replicates used in the microarray analysis.

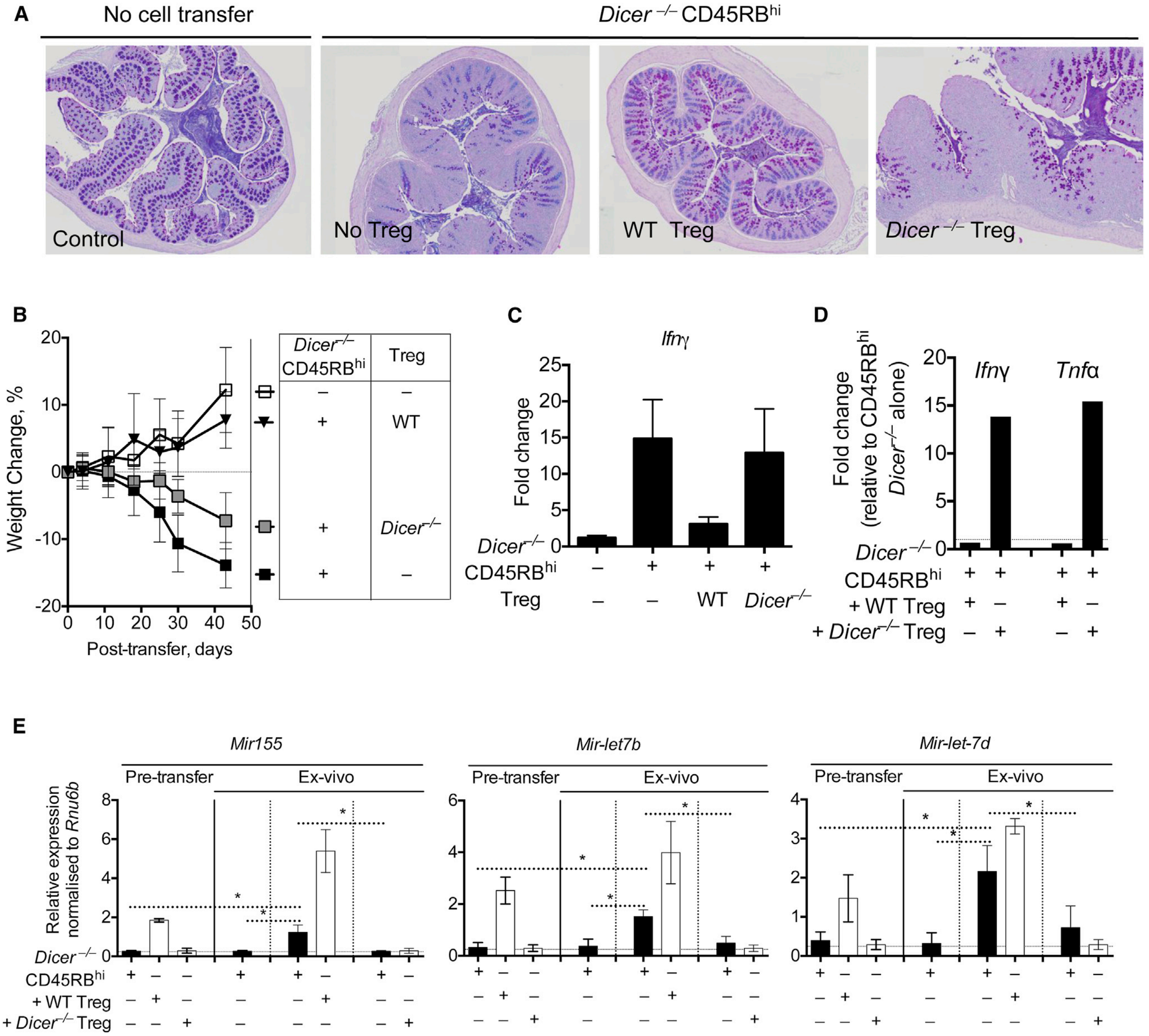

Figure 4. *Dicer*$^{-/-}$ Treg Cells Fail to Suppress Systemic Inflammation and Transfer miR-155, Let7-b, and Let-7d to Conventional T Cells In Vivo

Analysis of disease in mice after transfer of *Dicer*$^{-/-}$ CD4^{+}CD45RB^{hi} T cells with or without cotransfer of WT and *Dicer*$^{-/-}$ Treg (CD4^{+}CD25^{hi}) cells.

(A) Histopathology of colon sections stained with AB-PAS 5 weeks after cell transfer.

(B) Weight loss after *Dicer*$^{-/-}$ CD45RB^{hi} cell transfer alone or with WT or *Dicer*$^{-/-}$ Treg cells.

(C) Expression of *Ifng* in the colon of mice 5 weeks after cell transfer.

(D) Expression of *Ifng* and *Tnf* in ex vivo recovered *Dicer*$^{-/-}$ conventional T cells (CD4^{+}CD25^{-} eYFP^{+} *Dicer*$^{-/-}$) 5 weeks after cell transfer, as shown in Figure S4, from mice that received either WT (*Dicer*$^{-/-}$ + WT Treg cells) or *Dicer*$^{-/-}$ (*Dicer*$^{-/-}$ + *Dicer*$^{-/-}$ Treg cells) CD4^{+}CD25^{hi} Treg cells, mRNA expressed relative to *Dicer*$^{-/-}$ CD45RB^{hi} cell transfer alone. A representative of three experiments shown.

(E) Expression of *miR-155*, *Let-7b*, *Let-7d* in eYFP^{+}*Dicer*$^{-/-}$CD45Rb^{hi} cells, WT Treg cells, and *Dicer*$^{-/-}$ Treg cells before transfer (left three bars) or in ex vivo recovered, FACS-purified *Dicer*$^{-/-}$ effector T cell (CD4^{+}CD25^{-} eYFP^{+} *Dicer*$^{-/-}$) 5 weeks after cell transfer, from mice that received either *Dicer*$^{-/-}$ effector T cells alone, *Dicer*$^{-/-}$ effector T cells with WT Treg cells, or *Dicer*$^{-/-}$ conventional T cells with *Dicer*$^{-/-}$ Treg cells. *Dicer*$^{-/-}$ cells (black bars) or Treg cells (white bars). miRNA expression relative to *Rnu6b*. A representative of three experiments shown. *$p < 0.05$.

Error bars are SD.

inhibitor-treated Treg cells, which released Let-7d-depleted exosomes (Figure 7C), failed to prevent disease with significant weight loss, *Ifng* expression, and colonic inflammation (Figures 7F–7H). Collectively, these data support a mechanism of Treg-cell-mediated suppression through the release of miRNA-containing exosomes. Furthermore, we identified an important contribution of Let-7d in mediating this suppression in vitro and in vivo.

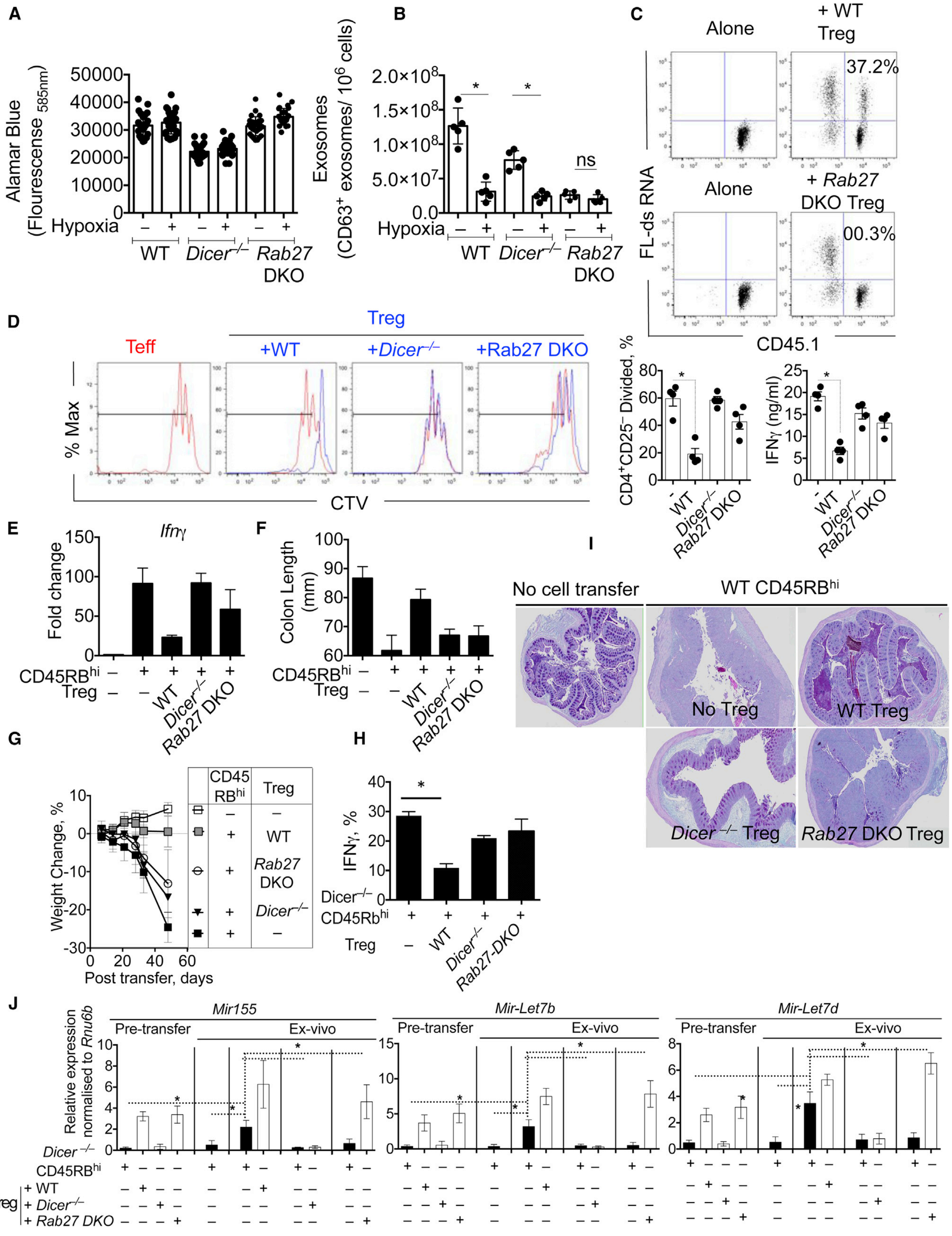

(legend on next page)

DISCUSSION

The control of immune responses is critical for survival, with compromised Treg-cell-mediated control resulting in multiorgan lymphocytic infiltration, organ failure, and death (Clark et al., 1999). In this study, we identified a mechanism of Treg-cell-mediated suppression via non-cell-autonomous gene regulation, mediated by miRNA-containing exosomes. Exosome-deficient, miRNA-deficient, or Let-7d-ablated Treg cells all failed to transfer miRNAs and failed to prevent lethal systemic inflammation. These data support a growing paradigm of non-cell-autonomous gene regulation previously reported in plants (Dunoyer et al., 2010) and animals (Sarkies and Miska, 2013; Valadi et al., 2007) and now demonstrated here, functioning as an important feature of immune regulation.

The mechanism of exosome release from Treg cells appears similar to other cells, with the exception of monensin treatment (Savina et al., 2003), which reduced exosome release from Treg cells, in contrast to exosome release from the K562 cell line. This might be due to the particularly sensitive nature of T cells to calcium changes (Wei et al., 1999) or might indicate that Golgi-mediated protein secretions support exosome release. Several overlapping pathways appear to regulate Foxp3 stability and exosome release, including inactivation of HIF-1α (Dang et al., 2011), ceramide biosynthesis (Kue et al., 2013), IL-2 signaling (Fontenot et al., 2005), and Amphiregulin signaling (Zaiss et al., 2013), suggesting that Foxp3 might regulate exosome release or that exosome-associated pathways might support Foxp3^{+} cells. In support of the latter, we identified that miR-155, which is involved in Treg cell development (Kohlhaas et al., 2009), was transferred from Treg cell to conventional T cell with the concomitant upregulation of several Treg-cell-associated genes in recipient cells (Figure 3).

Whether exosome contents are preferentially packaged within the parent cell or whether they are randomly assorted is not well understood. We previously identified significant miRNA heterogeneity between distinct Treg cell populations (Kelada et al., 2013) and found that such heterogeneity was influenced by the cytokine microenvironment. We report here that the miRNA content of Treg-cell-derived exosomes is distinct from the miRNA content of Th1- or Th2-cell-derived exosomes, indicating that cell/context-specific miRNAs may be packaged into exosomes. It is tempting to speculate that the local cytokine environment that shapes the miRNA profile of Treg cells (Kelada et al., 2013) also influences the exosome content of divergent T cells and that specific miRNA-containing exosomes are targeted to different effector cells. Here, we identified that Let-7d was preferentially packaged and transferred to Th1 cells, suppressing Th1 cell proliferation and IFN-γ secretion; whether different miRNAs are delivered to different Th cells is unclear.

Similarly, the rate of packaging and transfer of miRNAs between cells has not been elucidated. Recent reports indicate that at the immunological synapse between T cells and APCs, a microvesicle-rich pocket is formed (Choudhuri et al., 2014), providing a focused and protected region between cells, potentially allowing mass and rapid transfer of material. Whether miRNAs are transferred in these regions between T cell and APC was unclear from this study. However, for T cell-to-T cell transfer, the precise mechanism of exosome delivery is yet to be clarified. Furthermore, the precise mechanism of exosome uptake by recipient cell remains largely unknown.

Regulatory T cells have a growing repertoire of means to inhibit and prevent immune cell activation (Shevach, 2009). With respect to suppression of conventional T cells, current mechanisms include short-range, cell-contact-dependent (cytolysis and inhibitory receptor engagement) and potentially longer-range, cell-contact-independent (IL-2 consumption and suppressive cytokine secretions, such as IL-10, TGF-β, and IL-35) mechanisms. However, a combination of cell contact and secreted mechanisms is most likely optimal for Treg-cell-mediated suppression. From this study we can add

Figure 5. The GTPase Rab27 Is Needed for Treg Cell Exosome Secretion and Is Essential for Treg Cell Function In Vitro and In Vivo

Analysis of Treg cell function in vitro and in vivo after transfer of CD4^{+}CD45RB^{hi} T cells with or without transfer of CD4^{+}CD25^{hi} WT, *Dicer*$^{-/-}$, or *Rab27*-DKO Treg cells.

(A) WT, *Dicer*$^{-/-}$, or *Rab27*-DKO Treg cells were stimulated on anti-CD3 (1 μg/ml)- and anti-CD28 (5 μg/ml)-coated plates for 3 days. Cell viability/proliferation was determined by Alamar blue fluorescence intensity in culture supernatants.

(B) Exosomes were purified from the supernatant of stimulated WT, *Dicer*$^{-/-}$, or *Rab27*-DKO Treg cells, as in Figure 1.

(C) CD45.2^{+} WT Treg and CD45.2^{+} *Rab27*-DKO Treg cells were transfected with FL-dsRNA and cocultured naive WT CD45.1^{+} conventional Teff cells (CD4^{+}CD25^{-}CD44^{-}) on anti-CD3 (1 μg/ml)- and anti-CD28 (5 μg/ml)-coated plates. After 24 hr, cells were stained for CD45.1 and analyzed for FL-dsRNA by FACS.

(D) Proliferation of violet-labeled in vitro generated Th1 cells cultured alone (red trace in all four plots) or in presence of WT, *Dicer*$^{-/-}$, or *Rab27*-DKO Treg cells (blue trace) for 3 days. Summarized in bar chart showing the percentage of divided cells in the presence of the indicated Treg cell population. IFN-γ measured in the supernatant of cocultured cells.

(E) *Rag2*$^{-/-}$ mice were given CD4^{+}CD45RB^{hi} T cells with or without transfer of CD4^{+}CD25^{hi} WT, *Dicer*$^{-/-}$, or *Rab27*-DKO Treg cells. Expression of *Ifng* in the colon of mice 5 weeks after cell transfer.

(F) Colon length was measured 5 weeks after cell transfer.

(G) Weekly weight measurements after cell transfer, as in (E).

(H) Percentage of IFN-γ^{+} cells in the mesenteric lymph nodes 5 weeks after cell transfer.

(I) Histopathology of large intestine 5 weeks after cell transfer.

(J) *Rag2*$^{-/-}$ mice were given *Dicer*$^{-/-}$ CD4^{+}CD45RB^{hi} T cells with or without transfer of CD4^{+}CD25^{hi} WT, *Dicer*$^{-/-}$, or *Rab27*-DKO Treg cells. Expression of *miR-155*, *Let-7b*, *Let-7d* in eYFP^{+}*Dicer*$^{-/-}$CD45Rb^{hi} cells, WT Treg cells, *Dicer*$^{-/-}$ Treg cells, and *Rab27*-DKO Treg cells before transfer (left four bars) or in ex vivo recovered, FACS-purified *Dicer*$^{-/-}$ conventional T cells (CD4^{+}CD25^{-} eYFP^{+} *Dicer*$^{-/-}$) 5 weeks after cell transfer, from mice that received either *Dicer*$^{-/-}$ effector T cells alone, *Dicer*$^{-/-}$ conventional T cells with WT Treg, *Dicer*$^{-/-}$ effector T cells with *Dicer*$^{-/-}$ Treg cells, or *Dicer*$^{-/-}$ conventional T cells with *Rab27*-DKO Treg cells. *Dicer*$^{-/-}$ cells (black bars) or Treg cells (white bars). miRNA expression relative to *Rnu6b*. A representative of at least three experiments shown. *$p < 0.05$.

Error bars are SD.

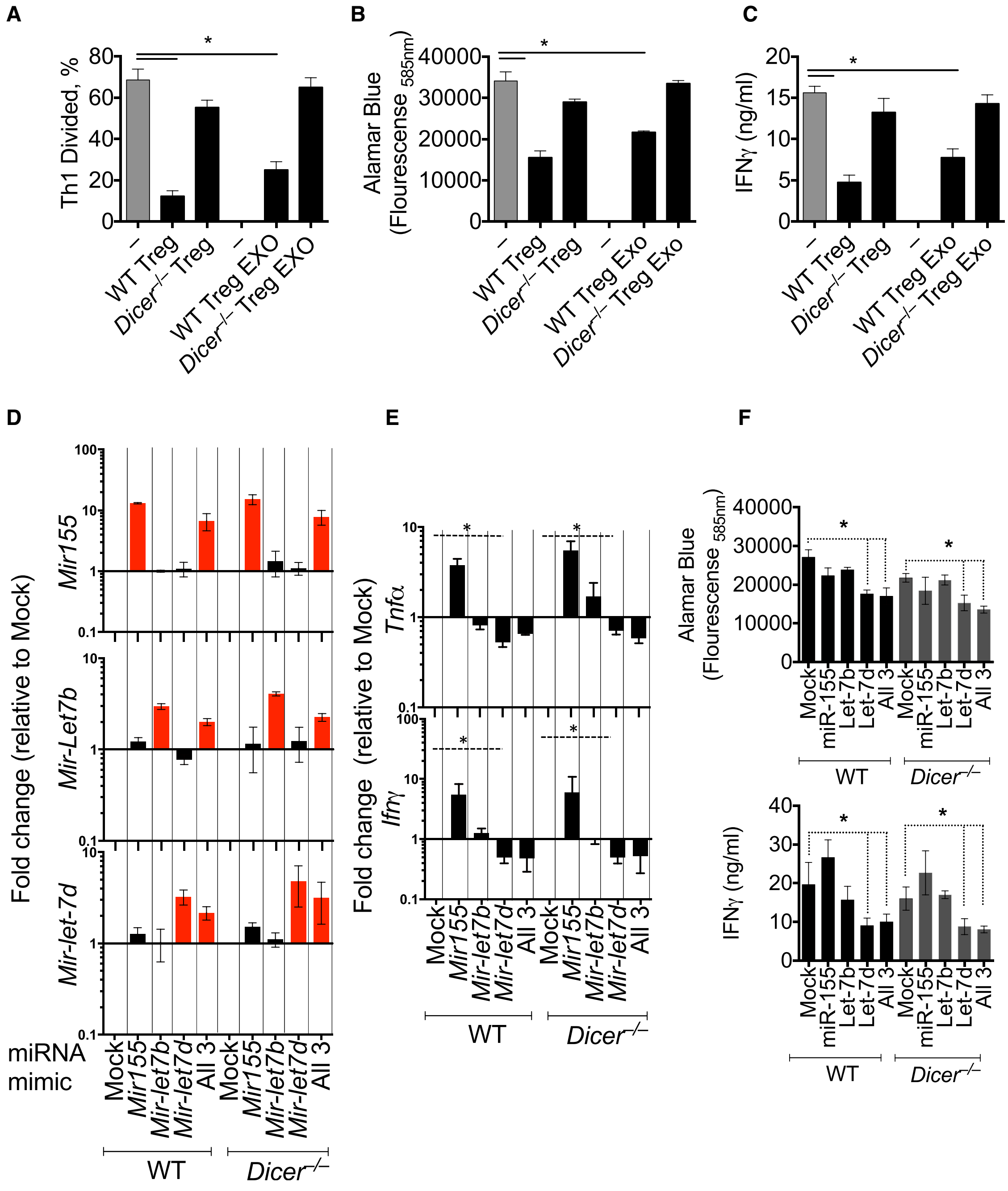

Figure 6. Treg-Cell-Derived Exosomes and Exogenous Let-7d Suppress Th1 Cell Proliferation and IFN-γ Secretion

(A–C) Cell trace violet-labeled Th1 cells were cultured with WT or *Dicer*$^{-/-}$ Treg cells, as in Figure 5D, or with exosomes recovered from WT or *Dicer*$^{-/-}$ Treg cells.

(A) Cell proliferation was determined by cell trace violet dilution.

(B) Alamar blue fluorescence intensity was determined in culture supernatants.

(C) IFN-γ secretion determined was determined in culture supernatants by ELISA.

Th1 cells were transfected with miR-155, Let-7b, Let-7d miRNA mimics (100 nM), as indicated.

(D) miR-155, Let-7b, and Let-7d were measured in Th1 cells, 24 hr after transfection with miR-155, Let-7b, Let-7d miRNA mimics. miRNAs are expressed relative to mock-transfected cells.

(E) *Tnf* and *Ifng* expression was measured in Th1 cells, 24 hr after transfection with miR-155, Let-7b, Let-7d miRNA mimics. mRNAs are expressed relative to mock-transfected cells.

(F) Proliferation (top) of transfected cells was determined by Alamar blue fluorescence tin culture supernatants. IFN-γ secretion (bottom) was determined by ELISA. A representative of three experiments shown.

*$p < 0.05$. Error bars are SD.

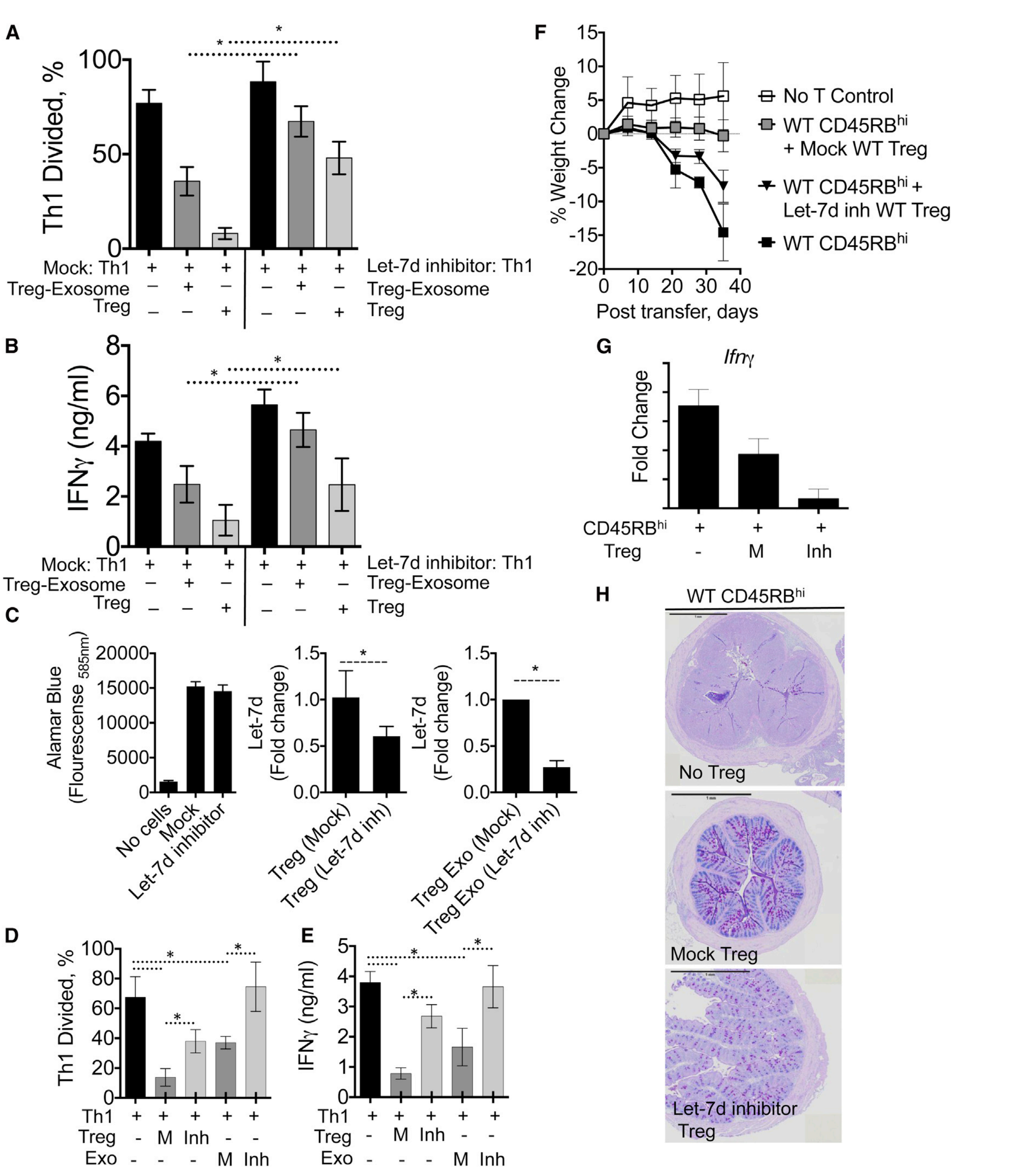

Figure 7. Let-7d from Treg Cells or Treg-Cell-Derived Exosomes Is Required to Suppress Th1 Cell Responses

(A–C) Th1 cells were transfected with Let-7d miRNA short hairpin inhibitors (100 nM), or mock transfected as indicated and cocultured with Treg cells or Treg-cell-derived exosomes.

(A) Proliferation of violet cell trace-labeled Th1 cells cultured alone or in presence of Treg cells or Treg-cell-derived exosomes for 3 days.

(B) IFN-γ measured in the supernatant of cocultured cells.

(C) Freshly isolated WT Treg cells were transfected with Let-7d miRNA short hairpin inhibitors (100 nM), or mock transfected as indicated, with cell proliferation determined by Alamar blue (left) and Let-7d expression analyzed in Treg cells (middle) or in Treg-cell-derived exosomes (right).

(D) Violet cell trace-labeled Th1 cells were cocultured with mock (M)- or Let-7d inhibitor-transfected Treg cells (Inh) or with exosomes isolated from Mock (M)- or Let-7d inhibitor-transfected Treg cells (Inh), as indicated. Proliferation of violet cell trace-labeled Th1 cells was determined after 3 days. Summarized in bar chart showing the percentage of divided cells in each condition.

(E) IFN-γ measured in the supernatant of cocultured cells, as in (D).

(F–H) Analysis of disease in mice at week 5 after transfer of WT $CD4^+CD45RB^{hi}$ T cells with or without mock-transfected or Let-7d inhibitor-transfected Treg cells. 10^5 Treg cells were adoptively transferred on week 2, 3, and 4.

(legend continued on next page)

non-cell-autonomous gene silencing as a mode of cell-contact-independent Treg-cell-mediated suppression, via transfer of miRNAs. We observed that isolated Treg-cell-derived exosomes can suppress conventional T cells; however, this was not as efficient as Treg cells, indicating that exosome-mediated transfer and additional mechanisms are indeed required for optimal suppression. Although cell contact was not required for the transfer of RNA between cells, whether cell contact allows more rapid and focused transfer, as mentioned above, is currently under investigation. Together these observations suggest that exosome-mediated transfer of miRNAs may collaborate with other Treg cell mechanisms for optimal suppression. For example, Treg cells anchored to other T cells via galectins (Garín et al., 2007) or to APCs via CTLA-4 might be required for most efficient delivery of miRNA-containing exosomes to target cells. From data presented here, the ability of Treg cells to release microvesicles in a Rab27-dependent manner, and specifically transfer Let-7d to target cells, was required for optimal Treg-cell-mediated suppression in vitro and in vivo. Whether this mechanism works in concert with other previously described Treg-cell-associated mechanisms or not is currently unclear.

Of the many potential Let-7d targets in recipient Th1 cells, we identified *Ptgs2* (Cox-2) as a credible candidate (Figures S6 and S7). Cox-2 is involved in Th1-cell-mediated responses and is responsible for lethal T-cell-mediated inflammation (Brewer et al., 2003; Iñiguez et al., 1999). Whether Treg-cell-derived Let-7d-containing exosomes directly target Cox-2 in Th cells, analogous to a “Cox-2 inhibitor,” is currently unclear. Supporting this pathway we observed reduced expression of Cox-2 and other Th1-cell-associated genes in suppressed T cells in vitro, concomitant with the acquisition of Let-7d from WT Treg cells. Furthermore, treatment of T cells with a COX-2 inhibitor reduced IFN-γ production by Th1 cells (Figure S6J). Given that Cox-2 inhibitors can prevent Th1-cell-mediated experimental autoimmunity (Muthian et al., 2006; Ni et al., 2007) and colitis (Paiotti et al., 2012; Zrieki et al., 2010), targeting of Cox-2 by Treg-cell-derived Let-7d may be a specific Treg-cell-mediated function to prevent lethal Th1-cell-mediated inflammation. In summary, this mechanism of Treg-cell-mediated suppression opens up the possibility that Treg cells package and deliver different proteins and RNA species, including miRNAs as we report here, to different cells at different times in a context-dependent manner.

EXPERIMENTAL PROCEDURES

Animals

Female C57BL/6, *Rag2*$^{-/-}$, CD45.1 *Cd4*^{Cre}*Dicer*$^{fl/fl}$ *R26*^{eyfp} (*Dicer*$^{-/-}$), *Rab27a*$^{ashen/ashen}$*b*$^{-/-}$ (*Rab27*-DKO), *miR155*$^{-/-}$, *Il4*^{gfp}*Ifng*^{yfp}*Il17a*^{Cre} (Hirota et al., 2013), *R26*^{eFP635}, *Foxp3*^{rfp}, and *Foxp3*^{gfp} 6- to 8-week-old (or as indicated) animals were bred and kept in the specific-pathogen-free facility at the Medical Research Council, NIMR. All animal experiments were performed according to institutional guidelines and the UK Home Office regulations. A minimum of five mice per group was used in each experiment, unless indicated.

Induction of Colitis and Systemic Inflammation

Rag2$^{-/-}$ mice were injected i.v. with 5×10^5 FACS-purified CD4$^+$CD45RB^{hi} T cells with or without 10^5 CD4$^+$CD25$^+$ Treg cells 2 weeks later, as previously described (Powrie et al., 1994).

In Vitro Cell Culture, Reagents, and Stimulation Assays

Primary cells were isolated from naive or diseased spleen, lymph nodes, or tissue, as indicated, by mechanical disruption. Full details of antibodies used can be found in Supplemental Experimental Procedures. For suppression assays, 10^4 Teff cells were labeled with cell trace violet (Invitrogen) as per manufacturer’s guidelines and stimulated with plate-bound anti-CD3 (1 μg/ml) and soluble CD28 (5 μg/ml) for 3 days in the presence or absence of Treg cells, at the indicated ratios.

RNA Extraction, RT-PCR, and Microarray

FACS-purified cells or isolated exosomes were stored in RLT lysis buffer at –80°C until RNA was extracted using RNeasy mini spin columns (QIAGEN). For RT-PCR, miScript RT or Quantitect RT was performed, according to manufacturer’s recommendations (QIAGEN). Real-time RT-PCR was performed on an ABI Prism 7900HT Sequence Detection System (Applied Biosystems) with relative quantities of mRNA and miRNA determined using SYBR Green PCR Master Mix (Applied Biosystems) and by the comparative threshold cycle method as described by Applied Biosystems for the ABI Prism 7700/7900HT Sequence Detection Systems. mRNA levels were normalized to HPRT and miRNA levels were normalized to RNU6B and then expressed as a relative increase or decrease compared with levels in controls, or as indicated.

ELISA

IFN-γ was measured by ELISA using cytokine capture and biotinylated detection antibodies (R&D Systems). The concentration of IFN-γ was determined from a serial-fold diluted standard curve with OD read at 450 nm in an ELISA reader.

miRNA Mimic and Hairpin Inhibitor Transfection

Cells were transfected with 100 nM of FL-dsRNA (SiGlo, Thermo Scientific Dharmacon) or BloCKiT (Invitrogen), miR-155 mimics, Let-7d mimics or hairpin inhibitors, Let-7b mimics, Cel-miR-67 hairpin inhibitors, or Cel-miR-239b hairpin inhibitors (Thermo Scientific Dharmacon) or MOCK transfected using Nucelofection reagents according to manufacturer’s recommendations (Amaxa).

Exosome Isolation and Analysis

Throughout these studies, complete media with exosome-depleted FCS (following 100,000 × *g* centrifugation) was used (Thery et al., 2006). Exosomes were purified by a combination of ultracentrifugation and using Exoquick solution (SBI System Bioscience) (Taylor et al., 2011), as previously reported and tested (King et al., 2012).

Full details of exosome isolation and other methods can be found in the Supplemental Experimental Procedures.

ACCESSION NUMBERS

The microarray data are available in the Gene Expression Omnibus (GEO) database (http://www.ncbi.nlm.nih.gov/gds) under the accession number GSEXXXX.

SUPPLEMENTAL INFORMATION

Supplemental Information includes seven figures, three tables, and Supplemental Experimental Procedures and can be found with this article online at http://dx.doi.org/10.1016/j.immuni.2014.05.019.

(F) Weight loss measured weekly.
(G) *Ifng* expression measured in the colon at week 5 after cell transfer.
(H) Histopathology of large intestine 5 weeks after cell transfer. A representative of three experiments shown.
*$p < 0.05$. Error bars are SD.

AUTHOR CONTRIBUTIONS

M.S.W. initiated the project, carried out most of the cell biological experiments, and contributed to the experimental design and data interpretation. I.S.O., V.S.P, S.M.C., S.C., and V.P. carried out experiments and helped with transfections. T.T. and M.C.S. provided animals, helped with interpretation of experiments, and provided input for the manuscript. M.S.W supervised the project and wrote the manuscript with input from the other authors and readers.

ACKNOWLEDGMENTS

We are grateful to A. O'Garra, V. Tybulewicz, B. Stockinger, and G. Kassiotis for critically reading the manuscript; to D. Anastasiou for help with hypoxia experiments; to A. Sessay, H. Jani, and L. Bhaw-Rosun in the Systems biology department for help with microarray experiments; to R. Mahmood and R. Anand for help with histology; to S. Kelada, Chapel Hill, UNC, for help with microarray analysis; to A. Potocnik for *R26*^{eFP635} mice; to G. Preece, W. Turnbull, and Bhavik Patel for assistance with flow cytometry-related work; to T. Norton, K. Williams, A. Adekoya, and B2, Dunkin, and building C staff for animal husbandry; and to J. Perez-Lloret for assistance with experiments. This work was supported by the Medical Research Council (MRC File Reference number MC_UP_A253_1028).

Received: February 13, 2014
Accepted: May 15, 2014
Published: July 17, 2014

REFERENCES

Alonso, M.A., and Millán, J. (2001). The role of lipid rafts in signalling and membrane trafficking in T lymphocytes. J. Cell Sci. *114*, 3957–3965.

Banerjee, A., Schambach, F., DeJong, C.S., Hammond, S.M., and Reiner, S.L. (2010). Micro-RNA-155 inhibits IFN-gamma signaling in CD4+ T cells. Eur. J. Immunol. *40*, 225–231.

Bolasco, G., Tracey-White, D.C., Tolmachova, T., Thorley, A.J., Tetley, T.D., Seabra, M.C., and Hume, A.N. (2011). Loss of Rab27 function results in abnormal lung epithelium structure in mice. Am. J. Physiol. Cell Physiol. *300*, C466–C476.

Boyman, O., Kovar, M., Rubinstein, M.P., Surh, C.D., and Sprent, J. (2006). Selective stimulation of T cell subsets with antibody-cytokine immune complexes. Science *311*, 1924–1927.

Brewer, J.A., Khor, B., Vogt, S.K., Muglia, L.M., Fujiwara, H., Haegele, K.E., Sleckman, B.P., and Muglia, L.J. (2003). T-cell glucocorticoid receptor is required to suppress COX-2-mediated lethal immune activation. Nat. Med. *9*, 1318–1322.

Bryniarski, K., Ptak, W., Jayakumar, A., Püllmann, K., Caplan, M.J., Chairoungdua, A., Lu, J., Adams, B.D., Sikora, E., Nazimek, K., et al. (2013). Antigen-specific, antibody-coated, exosome-like nanovesicles deliver suppressor T-cell microRNA-150 to effector T cells to inhibit contact sensitivity. J. Allergy Clin. Immunol. *132*, 170–181.

Cai, C.K., Zhao, G.Y., Tian, L.Y., Liu, L., Yan, K., Ma, Y.L., Ji, Z.W., Li, X.X., Han, K., Gao, J., et al. (2012). miR-15a and miR-16-1 downregulate CCND1 and induce apoptosis and cell cycle arrest in osteosarcoma. Oncol. Rep. *28*, 1764–1770.

Choudhuri, K., Llodrá, J., Roth, E.W., Tsai, J., Gordo, S., Wucherpfennig, K.W., Kam, L.C., Stokes, D.L., and Dustin, M.L. (2014). Polarized release of T-cell-receptor-enriched microvesicles at the immunological synapse. Nature *507*, 118–123.

Clark, L.B., Appleby, M.W., Brunkow, M.E., Wilkinson, J.E., Ziegler, S.F., and Ramsdell, F. (1999). Cellular and molecular characterization of the scurfy mouse mutant. J. Immunol. *162*, 2546–2554.

Dang, E.V., Barbi, J., Yang, H.Y., Jinasena, D., Yu, H., Zheng, Y., Bordman, Z., Fu, J., Kim, Y., Yen, H.R., et al. (2011). Control of T(H)17/T(reg) balance by hypoxia-inducible factor 1. Cell *146*, 772–784.

Druz, A., Chu, C., Majors, B., Santuary, R., Betenbaugh, M., and Shiloach, J. (2011). A novel microRNA mmu-miR-466h affects apoptosis regulation in mammalian cells. Biotechnol. Bioeng. *108*, 1651–1661.

Dunoyer, P., Schott, G., Himber, C., Meyer, D., Takeda, A., Carrington, J.C., and Voinnet, O. (2010). Small RNA duplexes function as mobile silencing signals between plant cells. Science *328*, 912–916.

Fire, A., Xu, S., Montgomery, M.K., Kostas, S.A., Driver, S.E., and Mello, C.C. (1998). Potent and specific genetic interference by double-stranded RNA in *Caenorhabditis elegans*. Nature *391*, 806–811.

Fontenot, J.D., Rasmussen, J.P., Gavin, M.A., and Rudensky, A.Y. (2005). A function for interleukin 2 in Foxp3-expressing regulatory T cells. Nat. Immunol. *6*, 1142–1151.

Fukuda, M. (2013). Rab27 effectors, pleiotropic regulators in secretory pathways. Traffic *14*, 949–963.

Garín, M.I., Chu, C.C., Golshayan, D., Cernuda-Morollón, E., Wait, R., and Lechler, R.I. (2007). Galectin-1: a key effector of regulation mediated by CD4+CD25+ T cells. Blood *109*, 2058–2065.

Hirota, K., Turner, J.E., Villa, M., Duarte, J.H., Demengeot, J., Steinmetz, O.M., and Stockinger, B. (2013). Plasticity of Th17 cells in Peyer's patches is responsible for the induction of T cell-dependent IgA responses. Nat. Immunol. *14*, 372–379.

Hutvágner, G., McLachlan, J., Pasquinelli, A.E., Bálint, E., Tuschl, T., and Zamore, P.D. (2001). A cellular function for the RNA-interference enzyme Dicer in the maturation of the let-7 small temporal RNA. Science *293*, 834–838.

Iñiguez, M.A., Punzón, C., and Fresno, M. (1999). Induction of cyclooxygenase-2 on activated T lymphocytes: regulation of T cell activation by cyclooxygenase-2 inhibitors. J. Immunol. *163*, 111–119.

Josefowicz, S.Z., Lu, L.F., and Rudensky, A.Y. (2012). Regulatory T cells: mechanisms of differentiation and function. Annu. Rev. Immunol. *30*, 531–564.

Kelada, S., Sethupathy, P., Okoye, I.S., Kistasis, E., Czieso, S., White, S.D., Chou, D., Martens, C., Ricklefs, S.M., Virtaneva, K., et al. (2013). miR-182 and miR-10a are key regulators of Treg specialisation and stability during *Schistosome* and *Leishmania*-associated inflammation. PLoS Pathog. *9*, e1003451.

King, H.W., Michael, M.Z., and Gleadle, J.M. (2012). Hypoxic enhancement of exosome release by breast cancer cells. BMC Cancer *12*, 421.

Kohlhaas, S., Garden, O.A., Scudamore, C., Turner, M., Okkenhaug, K., and Vigorito, E. (2009). Cutting edge: the Foxp3 target miR-155 contributes to the development of regulatory T cells. J. Immunol. *182*, 2578–2582.

Kosaka, N., Iguchi, H., Yoshioka, Y., Takeshita, F., Matsuki, Y., and Ochiya, T. (2010). Secretory mechanisms and intercellular transfer of microRNAs in living cells. J. Biol. Chem. *285*, 17442–17452.

Kue, C.S., Lim, H.X., Jung, M.Y., Hong, H.J., Cho, D., and Kim, T.S. (2013). C6-ceramide in combination with transforming growth factor-β enhances Treg cell differentiation and stable FoxP3 expression in vitro and in vivo. Immunobiology *218*, 952–959.

Liston, A., Lu, L.F., O'Carroll, D., Tarakhovsky, A., and Rudensky, A.Y. (2008). Dicer-dependent microRNA pathway safeguards regulatory T cell function. J. Exp. Med. *205*, 1993–2004.

Miranda, K.C., Huynh, T., Tay, Y., Ang, Y.S., Tam, W.L., Thomson, A.M., Lim, B., and Rigoutsos, I. (2006). A pattern-based method for the identification of MicroRNA binding sites and their corresponding heteroduplexes. Cell *126*, 1203–1217.

Mittelbrunn, M., Gutiérrez-Vázquez, C., Villarroya-Beltri, C., González, S., Sánchez-Cabo, F., González, M.A., Bernad, A., and Sánchez-Madrid, F. (2011). Unidirectional transfer of microRNA-loaded exosomes from T cells to antigen-presenting cells. Nat Commun *2*, 282.

Montecalvo, A., Larregina, A.T., Shufesky, W.J., Stolz, D.B., Sullivan, M.L., Karlsson, J.M., Baty, C.J., Gibson, G.A., Erdos, G., Wang, Z., et al. (2012). Mechanism of transfer of functional microRNAs between mouse dendritic cells via exosomes. Blood *119*, 756–766.

Muljo, S.A., Ansel, K.M., Kanellopoulou, C., Livingston, D.M., Rao, A., and Rajewsky, K. (2005). Aberrant T cell differentiation in the absence of Dicer. J. Exp. Med. *202*, 261–269.

Muthian, G., Raikwar, H.P., Johnson, C., Rajasingh, J., Kalgutkar, A., Marnett, L.J., and Bright, J.J. (2006). COX-2 inhibitors modulate IL-12 signaling through JAK-STAT pathway leading to Th1 response in experimental allergic encephalomyelitis. J. Clin. Immunol. *26*, 73–85.

Nathan, C. (2002). Points of control in inflammation. Nature *420*, 846–852.

Ni, J., Shu, Y.Y., Zhu, Y.N., Fu, Y.F., Tang, W., Zhong, X.G., Wang, H., Yang, Y.F., Ren, J., Wang, M.W., and Zuo, J.P. (2007). COX-2 inhibitors ameliorate experimental autoimmune encephalomyelitis through modulating IFN-gamma and IL-10 production by inhibiting T-bet expression. J. Neuroimmunol. *186*, 94–103.

Nolte-'t Hoen, E.N., Buermans, H.P., Waasdorp, M., Stoorvogel, W., Wauben, M.H., and 't Hoen, P.A. (2012). Deep sequencing of RNA from immune cell-derived vesicles uncovers the selective incorporation of small non-coding RNA biotypes with potential regulatory functions. Nucleic Acids Res. *40*, 9272–9285.

Nolting, J., Daniel, C., Reuter, S., Stuelten, C., Li, P., Sucov, H., Kim, B.G., Letterio, J.J., Kretschmer, K., Kim, H.J., and von Boehmer, H. (2009). Retinoic acid can enhance conversion of naive into regulatory T cells independently of secreted cytokines. J. Exp. Med. *206*, 2131–2139.

Ostrowski, M., Carmo, N.B., Krumeich, S., Fanget, I., Raposo, G., Savina, A., Moita, C.F., Schauer, K., Hume, A.N., Freitas, R.P., et al. (2010). Rab27a and Rab27b control different steps of the exosome secretion pathway. Nat. Cell Biol. *12*, 19–30, 1–13.

Paiotti, A.P., Ribeiro, D.A., Silva, R.M., Marchi, P., Oshima, C.T., Neto, R.A., Miszputen, S.J., and Franco, M. (2012). Effect of COX-2 inhibitor lumiracoxib and the TNF-α antagonist etanercept on TNBS-induced colitis in Wistar rats. J. Mol. Histol. *43*, 307–317.

Powrie, F., Leach, M.W., Mauze, S., Menon, S., Caddle, L.B., and Coffman, R.L. (1994). Inhibition of Th1 responses prevents inflammatory bowel disease in scid mice reconstituted with CD45RBhi CD4+ T cells. Immunity *1*, 553–562.

Raposo, G., Nijman, H.W., Stoorvogel, W., Liejendekker, R., Harding, C.V., Melief, C.J., and Geuze, H.J. (1996). B lymphocytes secrete antigen-presenting vesicles. J. Exp. Med. *183*, 1161–1172.

Sakaguchi, S. (2000). Regulatory T cells: key controllers of immunologic self-tolerance. Cell *101*, 455–458.

Sarkies, P., and Miska, E.A. (2013). Molecular biology. Is there social RNA? Science *341*, 467–468.

Savina, A., Furlán, M., Vidal, M., and Colombo, M.I. (2003). Exosome release is regulated by a calcium-dependent mechanism in K562 cells. J. Biol. Chem. *278*, 20083–20090.

Shevach, E.M. (2000). Regulatory T cells in autoimmmunity. Annu. Rev. Immunol. *18*, 423–449.

Shevach, E.M. (2009). Mechanisms of foxp3+ T regulatory cell-mediated suppression. Immunity *30*, 636–645.

Singh, R.K., Mizuno, K., Wasmeier, C., Wavre-Shapton, S.T., Recchi, C., Catz, S.D., Futter, C., Tolmachova, T., Hume, A.N., and Seabra, M.C. (2013). Distinct and opposing roles for Rab27a/Mlph/MyoVa and Rab27b/Munc13-4 in mast cell secretion. FEBS J. *280*, 892–903.

Smyth, L.A., Ratnasothy, K., Tsang, J.Y., Boardman, D., Warley, A., Lechler, R., and Lombardi, G. (2013). CD73 expression on extracellular vesicles derived from CD4+ CD25+ Foxp3+ T cells contributes to their regulatory function. Eur. J. Immunol. *43*, 2430–2440.

Sowinski, S., Jolly, C., Berninghausen, O., Purbhoo, M.A., Chauveau, A., Köhler, K., Oddos, S., Eissmann, P., Brodsky, F.M., Hopkins, C., et al. (2008). Membrane nanotubes physically connect T cells over long distances presenting a novel route for HIV-1 transmission. Nat. Cell Biol. *10*, 211–219.

Taylor, D.D., Zacharias, W., and Gercel-Taylor, C. (2011). Exosome isolation for proteomic analyses and RNA profiling. Methods Mol. Biol. *728*, 235–246.

Théry, C. (2011). Exosomes: secreted vesicles and intercellular communications. F1000 Biol Rep *3*, 15.

Théry, C., Duban, L., Segura, E., Véron, P., Lantz, O., and Amigorena, S. (2002). Indirect activation of naïve CD4+ T cells by dendritic cell-derived exosomes. Nat. Immunol. *3*, 1156–1162.

Thery, C., Amigorena, S., Raposo, G., and Clayton, A. (2006). Isolation and characterization of exosomes from cell culture supernatants and biological fluids. Curr. Protoc. Cell Biol. *Chapter 3*, 222.

Trajkovic, K., Hsu, C., Chiantia, S., Rajendran, L., Wenzel, D., Wieland, F., Schwille, P., Brügger, B., and Simons, M. (2008). Ceramide triggers budding of exosome vesicles into multivesicular endosomes. Science *319*, 1244–1247.

Valadi, H., Ekström, K., Bossios, A., Sjöstrand, M., Lee, J.J., and Lötvall, J.O. (2007). Exosome-mediated transfer of mRNAs and microRNAs is a novel mechanism of genetic exchange between cells. Nat. Cell Biol. *9*, 654–659.

Voinnet, O., and Baulcombe, D.C. (1997). Systemic signalling in gene silencing. Nature *389*, 553.

Wang, G.J., Liu, Y., Qin, A., Shah, S.V., Deng, Z.B., Xiang, X., Cheng, Z., Liu, C., Wang, J., Zhang, L., et al. (2008). Thymus exosomes-like particles induce regulatory T cells. J. Immunol. *181*, 5242–5248.

Wei, X., Tromberg, B.J., and Cahalan, M.D. (1999). Mapping the sensitivity of T cells with an optical trap: polarity and minimal number of receptors for Ca(2+) signaling. Proc. Natl. Acad. Sci. USA *96*, 8471–8476.

Yang, X., Yu, J., Yin, J., Xiang, Q., Tang, H., and Lei, X. (2012). MiR-195 regulates cell apoptosis of human hepatocellular carcinoma cells by targeting LATS2. Pharmazie *67*, 645–651.

Zaiss, D.M., van Loosdregt, J., Gorlani, A., Bekker, C.P., Gröne, A., Sibilia, M., van Bergen en Henegouwen, P.M., Roovers, R.C., Coffer, P.J., and Sijts, A.J. (2013). Amphiregulin enhances regulatory T cell-suppressive function via the epidermal growth factor receptor. Immunity *38*, 275–284.

Zhou, X., Jeker, L.T., Fife, B.T., Zhu, S., Anderson, M.S., McManus, M.T., and Bluestone, J.A. (2008). Selective miRNA disruption in T reg cells leads to uncontrolled autoimmunity. J. Exp. Med. *205*, 1983–1991.

Zitvogel, L., Regnault, A., Lozier, A., Wolfers, J., Flament, C., Tenza, D., Ricciardi-Castagnoli, P., Raposo, G., and Amigorena, S. (1998). Eradication of established murine tumors using a novel cell-free vaccine: dendritic cell-derived exosomes. Nat. Med. *4*, 594–600.

Zrieki, A., Farinotti, R., and Buyse, M. (2010). Cyclooxygenase-2 inhibitors prevent trinitrobenzene sulfonic acid-induced P-glycoprotein up-regulation in vitro and in vivo. Eur. J. Pharmacol. *636*, 189–197.

Immunity, Volume *41*

Supplemental Information

microRNA-Containing T-Regulatory-Cell-Derived Exosomes Suppress Pathogenic T Helper 1 Cells

Isobel S. Okoye, Stephanie M. Coomes, Victoria S. Pelly, Stephanie Czieso, Venizelos Papayannopoulos, Tanya Tolmachova, Miguel C. Seabra, and Mark S. Wilson

Supplementary Figure S1

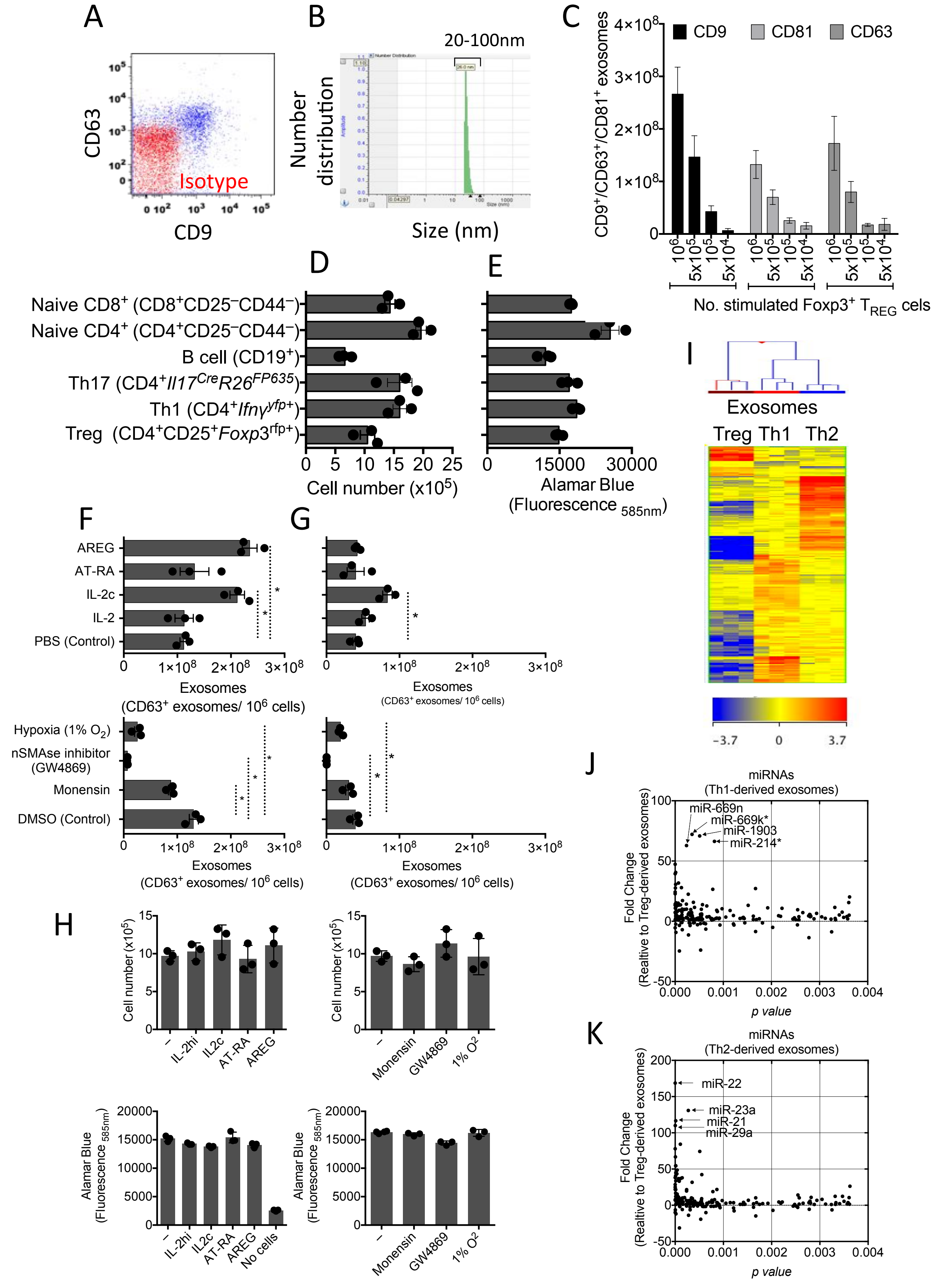

Supplementary Figure S2

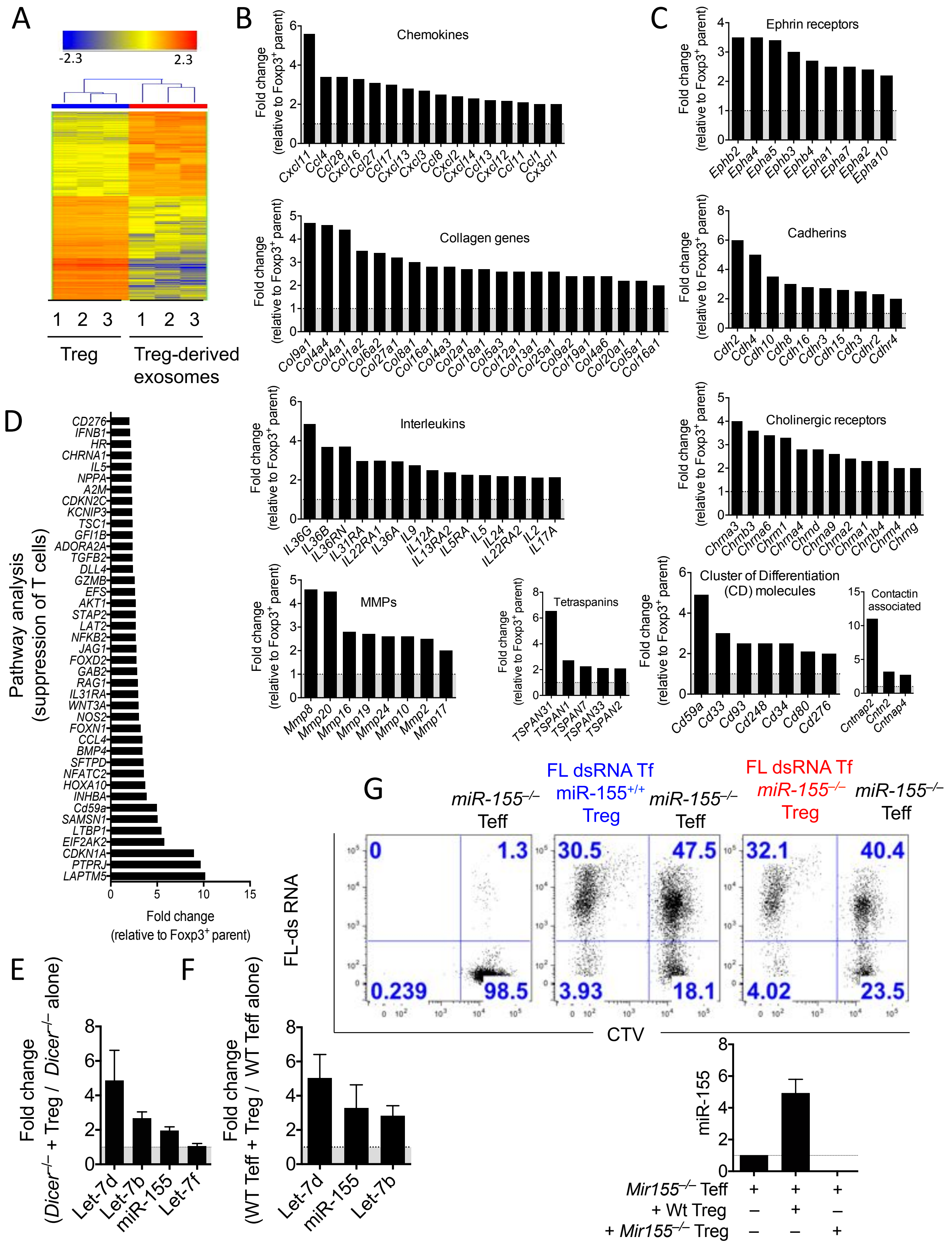

Supplementary Figure S3

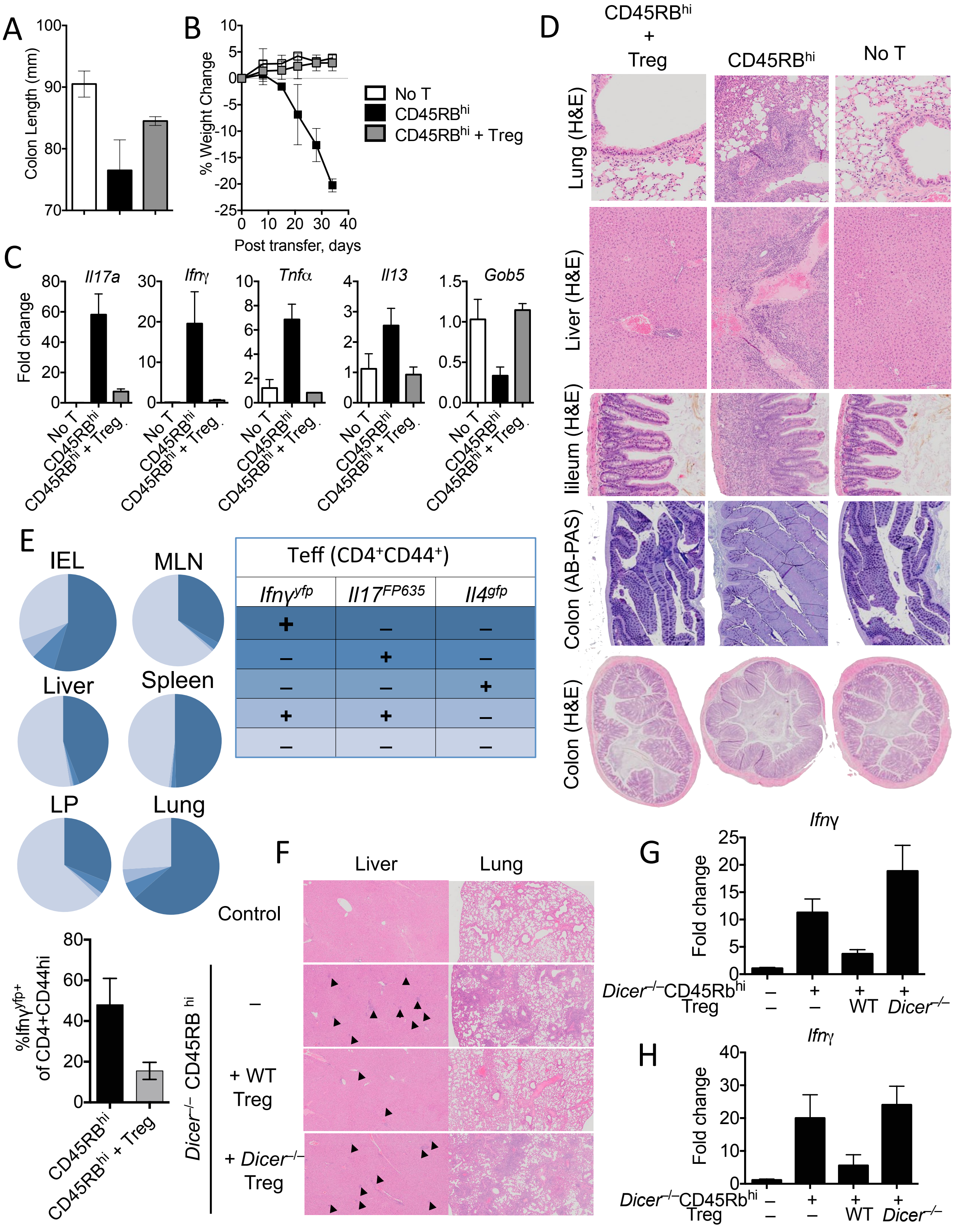

Supplementary Figure S4

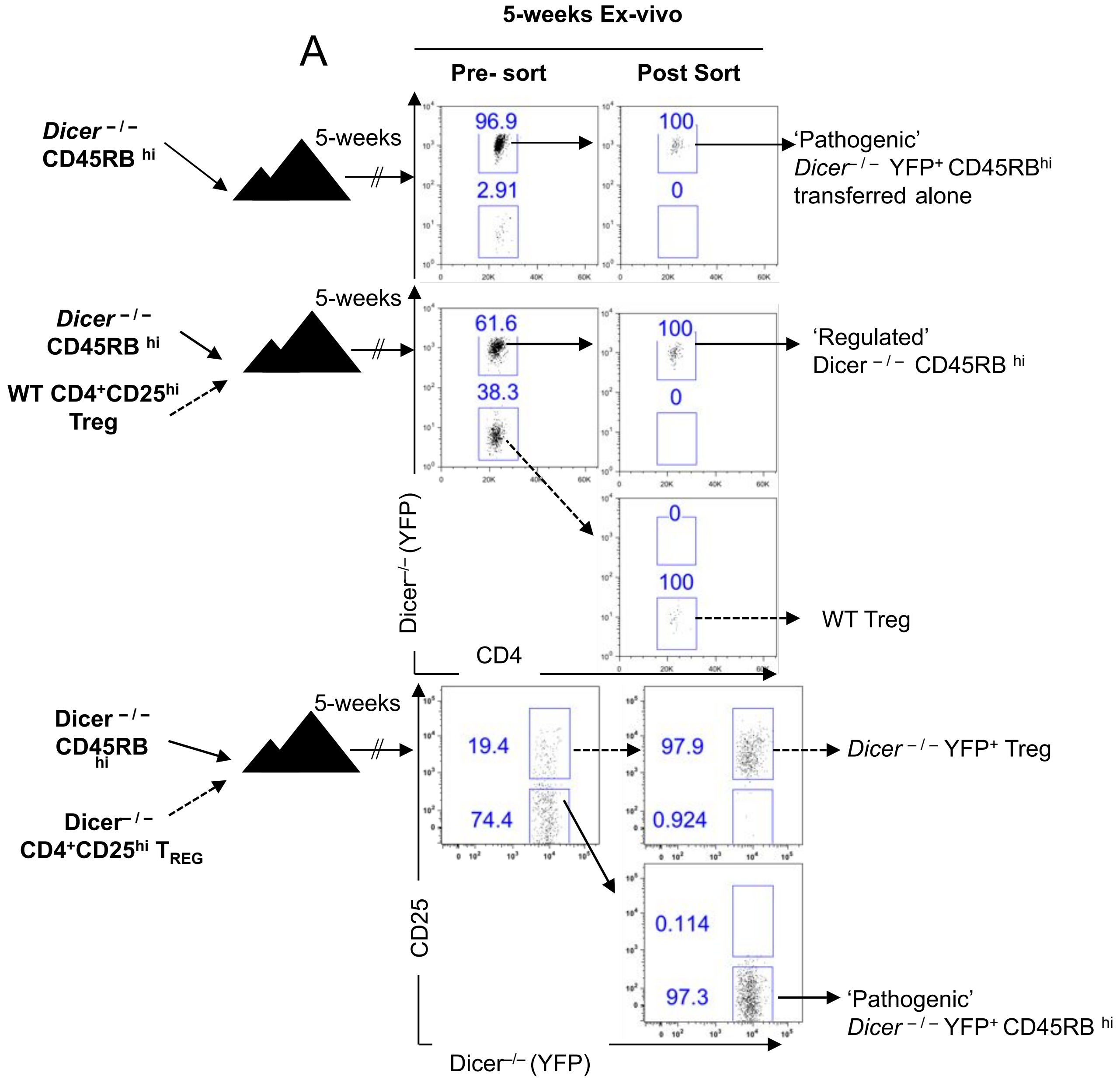

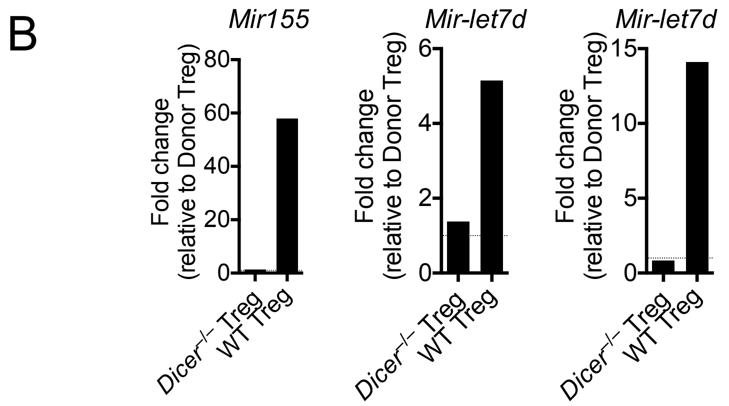

Supplementary Figure S5

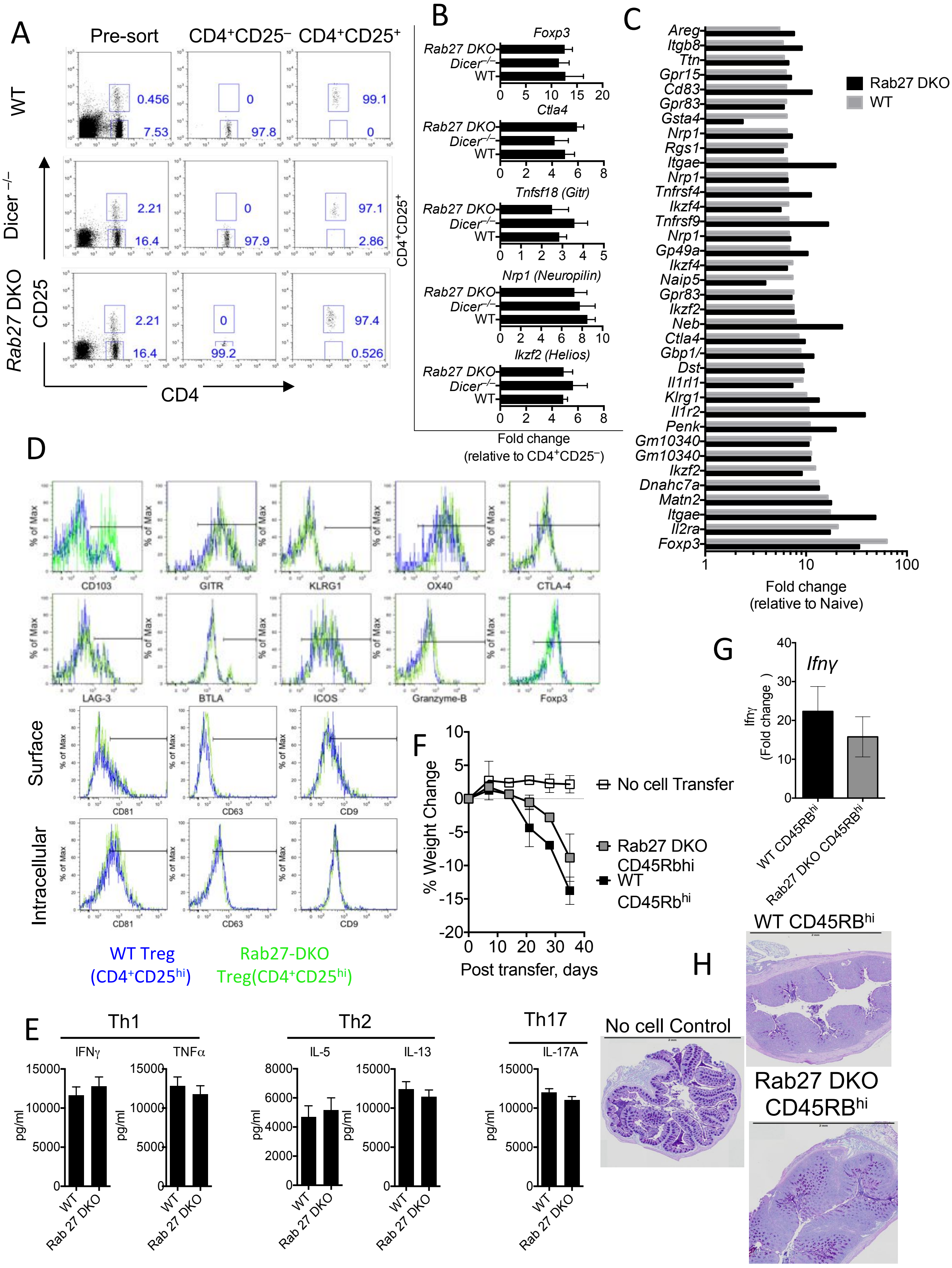

Supplementary Figure S6

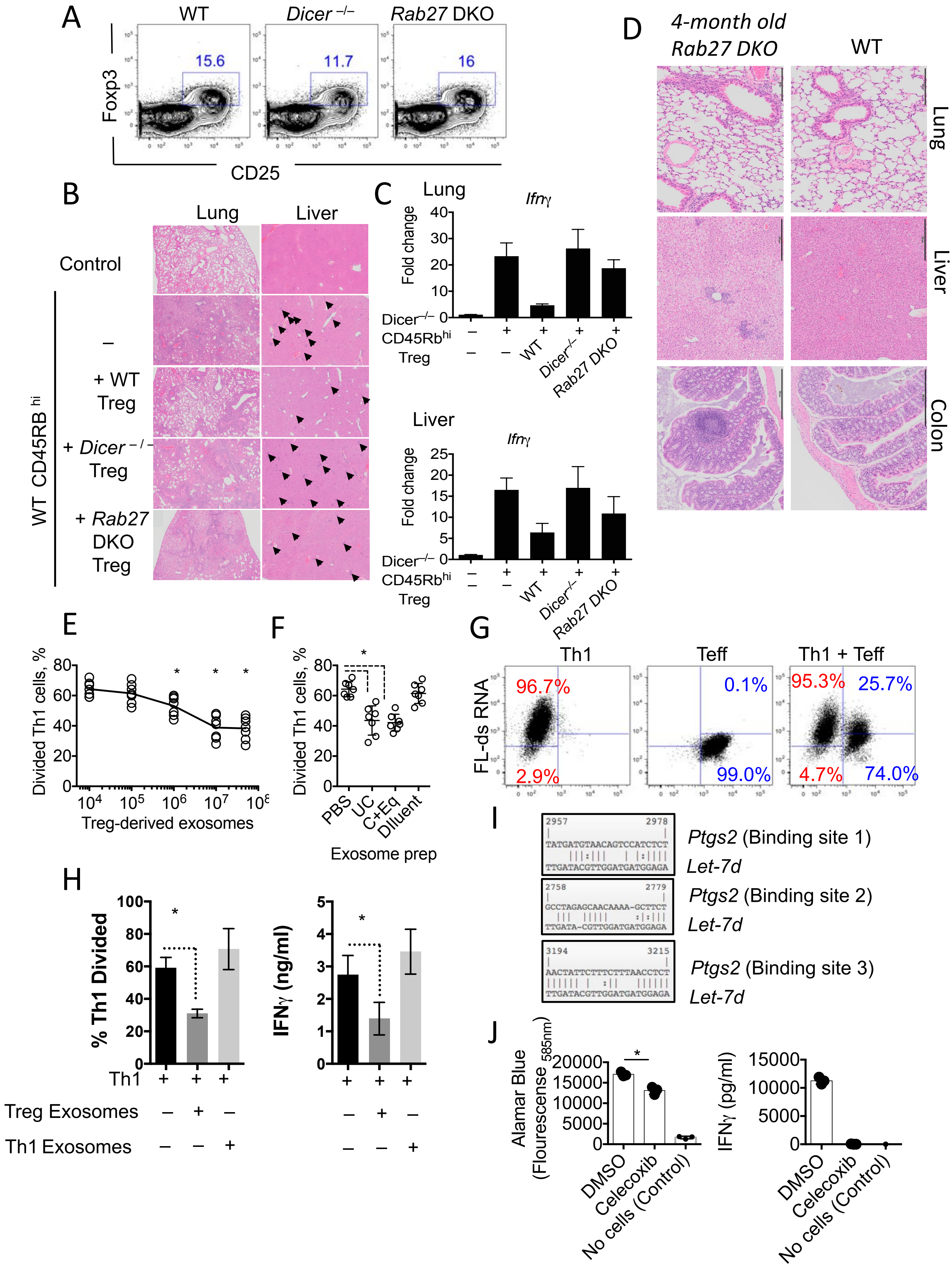

Supplementary Figure S7

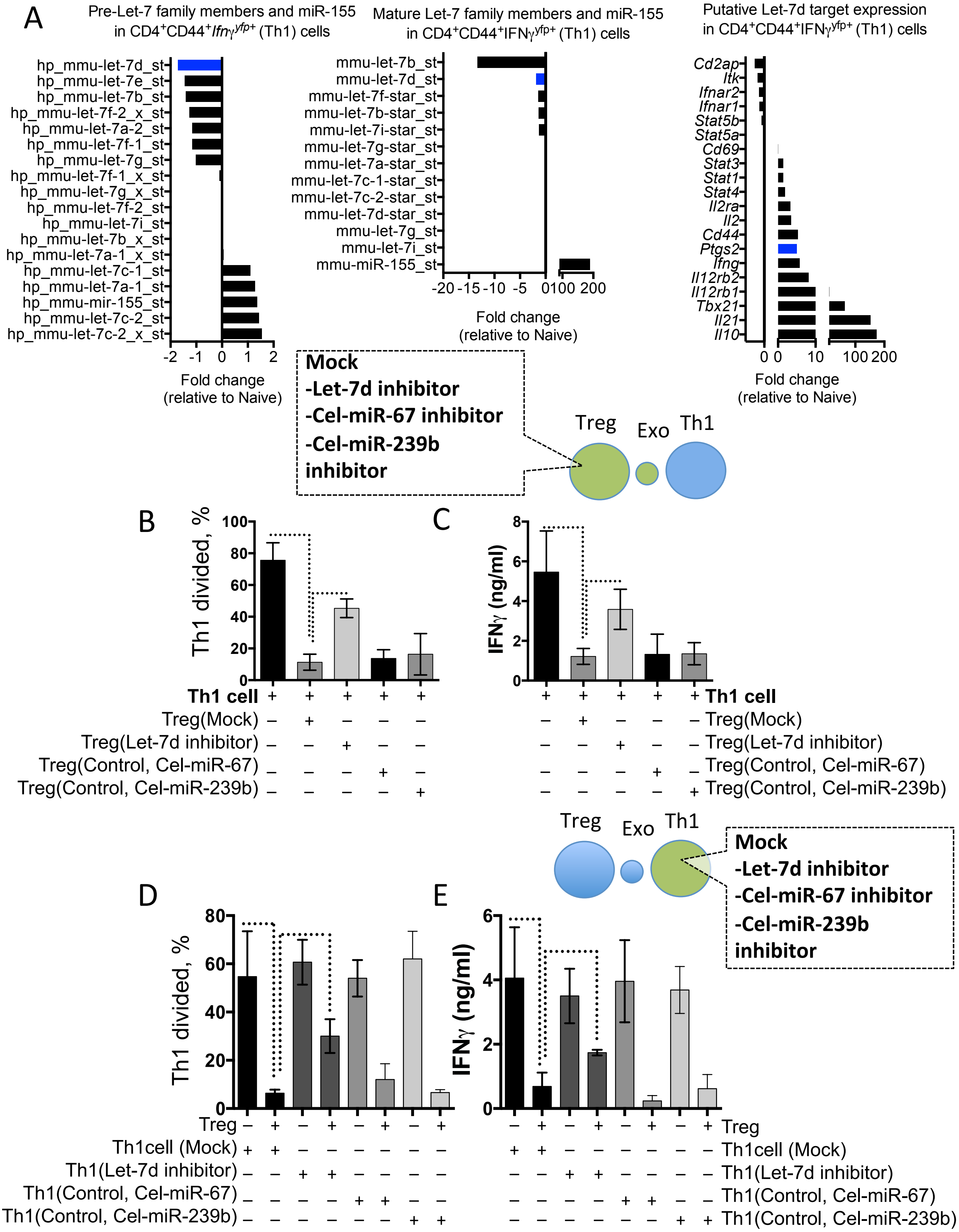

Supplementary Figures and Tables

Figure S1, related to Figure 1.

Treg- Th1 and Th2-derived exosome characteristics and cellular proliferation.

A, Treg cell derived exosomes were stained with anti-CD63 and anti-CD9 antibodies and analysed on a BD LSRII.

B, Purified exosomes were analysed using dynamic light scatter, giving a profile between 20-100nm in diameter.

C, Treg cell-derived exosomes were quantified using a CD9, CD81 or CD63 ELISA, with a known concentration of CD9, CD81 or CD63 used as a standard curve.

D-E, Following stimulation, as in Figure 1, cells were quantified by conventional trypan blue dye exclusion (left) and by Alamar blue fluorescence in culture supernatant (right).

F and G (top), Exosomes were quantified from the supernatant of Treg cells (**F**) or activated naïve cells (**G**) stimulated for 3 days with anti-CD3 (1μg/ml) and anti-CD28 (5μg/ml) with the addition of IL-2 (500ng/ml), IL-2c (5ng/ml of IL-2 in complex with 50μg/ml of anti-IL-2, JES6-1A2), AT-RA (10nM) or amphiregulin, (AREG, 100ng/ml), as indicated.

F and G, (bottom), Exosomes were quantified in the supernatant of Treg cells (**F**) or activated naïve T cells (**G**) cells cultured under normoxia (20% O_2), hypoxia (1% O_2), with a ceramide biosynthesis inhibitor (GW4869, 10μM) or monensin (10μM) for the last 6 hours of culture, as indicated. A representative of 3 experiments shown. * denotes $P<0.05$.

H, Treg cells were quantified by conventional trypan blue dye exclusion (top) and by Alamar blue fluorescence in culture supernatant (bottom) following the indicated treatments.

I, Treg cell-derived exosomes contain different miRNAs compared to Th1 or Th2 cell-derived exosomes. RNA was isolated from 3 biological replicates of Treg cell, Th1 cell or Th2 cell-derived exosomes and used for transcriptional analysis. Heat-map representing miRNA expression in Treg, Th1 cells or Th2 cells.

J, Dot plot showing miRNA expression in Th1- derived exosomes, relative to expression in Treg cell derived exosomes, with respective p value on x-axis.
K, Dot plot showing miRNA expression in Th2- derived exosomes, relative to expression in Treg cell derived exosomes, with respective p value on x-axis.

Figure S2, related to Figure 1.
mRNA content of Treg-derived exosomes and transfer of miRNAs from Treg to Teff cell.
A, RNA was isolated from 3 biological replicates of Treg cells and Treg-derived exosomes and used for transcriptional analysis. mRNA transcripts in Treg cell-derived exosomes are expressed relative to mRNA transcripts in Treg cells.
B-C, Expression of secreted (**B**) and surface (**C**) mRNA transcripts in Treg cell-derived exosomes are highlighted.
D, Ingenuity Pathways Analysis of differentially regulated mRNA transcripts in Treg-derived exosomes.
E, RNA was extracted from CD45.1^{+}*Cd4*^{Cre} *Dicer*$^{fl/fl}$ *R26*^{eyfp}CD4^{+}CD25^{-} Teff cells after being cultured alone or with WT Treg cells, as in **Figure 3**. Expression of Let-7b, d and f and miR-155 was measured by qRT-PCR.
F, RNA was extracted from WT CD45.1^{+}CD4^{+}CD25^{-} Teff cells after being cultured alone or with WT Treg cells, as in **Figure 3**. Expression of Let-7b, miR-155 and Let-7d was measured by qRT-PCR and expressed relative to WT Teff cells cultured alone.
G, CD45.1^{+} miR-155-sufficient Treg cells were co-cultured with CD45.2^{+} *miR-155*$^{-/-}$ Teff cells for 48 hours before being separated. RNA was extracted from purified CD45.2^{+} *miR-155*$^{-/-}$ Teff cells after co-culture and miR-155 was quantified by RT-PCR. A representative of 3 experiments shown. * denotes P<0.05.

Figure S3, related to Figure 4.
Treg cell-mediated suppression of systemic inflammation.
Analysis of disease in mice following transfer of *Dicer*$^{-/-}$ CD4^{+}CD45RB^{high} T cells with or without co-transfer of WT and *Dicer*$^{-/-}$ Treg (CD4^{+}CD25^{hi}) cells.
A, Colon length measured 5 weeks post cell transfer.

B, Weekly weight measurements following cell transfer.

C, RNA expression of several inflammatory and mucous-associated genes in the colon of mice 5 weeks post cell transfer.

D, Histopathology of lung, liver, small and large intestine 5-weeks post cell transfer.

E, Percentage of cytokine$^+$ cells ($Ifn\gamma^{yfp}$ $Il17a^{Cre}R26^{eFP635}$ $Il4^{gfp}$) in the intra-epithelial cells (IEL), mesenteric lymph nodes (MLN), liver, spleen, lamina propria (LP) and lung 5 weeks post cell transfer. A representative of 3 experiments shown. * denotes P<0.05.

F, Inflammatory foci in the liver and lung of mice 5-weeks post transfer of CD45RB^{hi}CD45.1$^+$*Cd4*^{Cre}*Dicer*$^{fl/fl}$ *R26*$^{eyfp+}$ cells alone or with WT or *Dicer*$^{-/-}$ Treg cells.

G and H, Expression of $Ifn\gamma$ in the liver (**G**) or lung (**H**) of mice 5-weeks post cell transfer. A representative of 3 experiments shown.

Figure S4, related to Figure 5.

Model of cell transfer and re-isolation.

A, Model and FACS plots of re-isolated ex-vivo *Dicer*$^{-/-}$ YFP$^+$CD4$^+$CD25$^-$ cells and Treg (YFP$^-$CD4$^+$CD25$^+$) cells, 5 weeks post cell transfer from mice that received either *Dicer*$^{-/-}$ CD45RB^{hi} cells alone ('Pathogenic'); *Dicer*$^{-/-}$ CD45RB^{hi} cells ('Regulated') with WT Treg cells; *Dicer*$^{-/-}$ CD45RB^{hi} cells with *Dicer*$^{-/-}$ Treg cells. *Dicer*$^{-/-}$ Treg were identified as YFP$^+$CD4$^+$CD25$^+$.

B, Expression of miR-155, Let-7b and Let-7d in WT and *Dicer*$^{-/-}$ Treg cells, isolated 5-weeks post cell transfer, expressed relative to donor WT or *Dicer*$^{-/-}$ Treg cells prior to transfer. A representative of 3 experiments shown.

Figure S5, related to Figure 5.

***Rab27*-DKO T cells (Treg and Th cells) are similar to WT T cells (Treg and Th cells).**

A, Treg (CD4$^+$CD25$^+$) and naïve T cells were FACS purified from WT, *Cd4*^{Cre}*Dicer*$^{fl/fl}$*R26*$^{eyfp+}$ and *Rab27-DKO* mice.

B, Expression of Treg -associated genes determined by qRT-PCR and expressed relative to naïve CD4$^+$CD25$^-$ T cells.

C, Expression of top 40 genes in WT and *Rab27-DKO* Treg cells, relative to naïve T cells.

D, Expression of surface (CD103, GITR, KLRG1, OX40, LAG-3, BTLA and ICOS) and intracellular (CTLA-4, Granzyme-B, Foxp3) Treg -associated proteins in WT (**Blue**) and *Rab27*-DKO (**Green**) Treg cells.

E, WT and *Rab27*-DKO Naïve T cells ($CD4^+CD44^-CD25^-$) were polarised under Th1, Th2 and Th17 conditions in vitro. Th1 (IFNγ, TNFα), T_H2 (IL-5 and IL-13) and T_H17 (IL-17A)-associated cytokines measured in cell culture supernatant after 7 days of culture.

F, Analysis of disease in *Rag2*$^{-/-}$ mice following transfer of WT or *Rab27*-DKO $CD4^+CD45RB^{high}$ T cells.

G, Colonic *Ifnγ* expression measured, 5-weeks post transfer.

H, Histopathology of large intestine 5-weeks post cell transfer. A representative of 2 experiments shown with 5 mice per group per experiment.

Figure S6, related to Figure 5.

***Rab27*-DKO Treg cells fail to prevent systemic T cell mediated disease. Treg-derived, but not Th1-derived, exosomes suppress Th1 cell proliferation and contain Let-7d which targets *Ptgs2* (Cox2).**

Analysis of Treg function in vivo following transfer of $CD4^+CD45RB^{high}$ T cells with or without transfer of $CD4^+CD25^{hi}$ WT, *Dicer*$^{-/-}$ or *Rab27-DKO* Treg cells.

A, Percentage of $CD25^+Foxp3^+$ cells in the CD4 compartment of the mesenteric lymph nodes of mice 5-weeks post cell transfer.

B, Inflammatory foci in the lung and liver of mice 5-weeks post transfer of WT $CD45RB^{hi}$ cells alone or with WT, *Dicer*$^{-/-}$ or *Rab27-DKO* Treg cells.

C, Expression of *Ifnγ* in the lung (left) or liver (right) of mice 5-weeks post cell transfer.

D, Inflammatory foci in the lung, liver and large intestine of 4-month old *Rab27-DKO* mice, relative to a 4-month old WT mouse**.** A representative of 2 experiments shown.

E, Naive T cells were cultured under Th1 conditions for 3 days before labeling with cell trace violet and culturing with increasing numbers of fresh Treg-derived exosomes. Cell trace violet dilution was assessed after a further 3

days of culture with exosomes and soluble anti-CD3 (1μg/ml) and anti-CD28 (5μg/ml).

F, Treg cell-derived exosomes were isolated by 2 different methods. **UC**, Treg cell supernatant from stimulated Treg cells was centrifuged at 300g for 10 minutes. The supernatant was recovered for further centrifugation at 2,000g for a further 30 minutes. The resulting supernatant was further centrifuged at 10,000g for 30 minutes. Finally, this supernatant was used to pellet exosomes at 100’000g for 2 hours. **C+Eq,** exosomes were recovered from Treg cell supernatant by a combination of centrifugation and Exoquick isolation. Treg cell supernatant from stimulated Treg cells was centrifuged at 300g for 10 minutes. The supernatant was recovered for further centrifugation at 2,000g for a further 30 minutes. The resulting supernatant was further centrifuged at 10,000g for 30 minutes. Finally, instead of 100’000g, the supernatant was mixed with Exoquick, as per manufacturers recommendation. Exosomes were re-suspended in PBS. **Diluent**, cell-free supernatant was used in the C+Eq, method, as above, as a control for the Exoquick procedure. 10^7 Treg -derived exosomes from each method were added to cell trace violet-labeled Th1 cells. Cell trace violet dilution was assessed after a further 3 days of culture with exosomes and soluble anti-CD3 (1μg/ml) and anti-CD28 (5μg/ml). A representative of 2 experiments shown. * denotes P<0.05.

G, Th1 cells transfer RNA to Teff cells, but are not suppressive. Th1 cells were transfected with FL-dsRNA and co-cultured with untransfected CD45.1+ Teff cells. After 24 hours, co-cultured cells were analysed by flow cytometry.

H, Exosomes were purified from Treg or Th1 cell cultures, quantified and added (10^7) to cell trace violet-labeled Th1 cells. Cells were cultured for 3 days before analysis of cell division and IFNγ measurement in supernatant.

I, Let-7d is predicted to target many genes involved in Th1 cell differentiation and effector function, including *Ptgs2***.**

J, Th1 cells were cultured with a Cox-2 inhibitor (Celecoxib, 50μM) or DMSO for 3 days. Alamar blue fluorescence and IFNγ secretion in culture supernatant were determined on day 3. A representative of 2 experiments shown. * denotes P<0.05.

Figure S7, related to Figure 6 and 7.

miRNAs and mRNA in Th1 cells suggest that Let-7d targets Ptgs2. Let-7d inhibitor, but not control inhibitor, treated Treg or Th1 cells, compromises Treg-mediated suppression.

A, Naïve $CD4^{+}CD25^{-}CD44^{-}IFN\gamma^{yfp-}$ cells were polarized under Th1 conditions for 1 week before FACS sorting $IFN\gamma^{yfp+}$ cells. miRNA and mRNAs were determined in FACS purified cytokine^{+} Th1 $CD4^{+}IFN\gamma^{yfp+}$ cells by microarray.
B and C, Freshly isolated WT Treg cells were either MOCK transfected, transfected with Let-7d miRNA short hairpin inhibitors (100nM), or transfected with control, Cel-miR-67 or Cel-miR-239b short hairpin inhibitors (both at 100nM), as indicated. Violet cell trace-labeled Th1 cells were co-cultured at an equal ratio with treated Treg cells, as indicated. Proliferation of violet cell trace-labeled Th1 cells (**B**) and secreted IFNγ (**C**) was determined after 3 days of culture with soluble anti-CD3 (1μg/ml) and anti-CD28 (5μg/ml).
D and E, Violet cell trace-labeled Th1 cells were either MOCK transfected, transfected with Let-7d miRNA short hairpin inhibitors (100nM), or transfected with control, Cel-miR-67 or Cel-miR-239b short hairpin inhibitors (both at 100nM). Treated Th1 cells were co-cultured at an equal ratio with Treg cells, as indicated. Proliferation of violet cell trace-labeled Th1 cells (**D**) and secreted IFNγ (**E**) was determined after 3 days of culture with soluble anti-CD3 (1μg/ml) and anti-CD28 (5μg/ml). A representative of 2 experiments shown. * denotes $P<0.05$.

Supplementary Tables

Table S1, related to Figure 1.

miRNA transcripts in Treg-derived exosomes.

miRNAs identified in Treg cell-derived exosomes with fold change of miRNAs relative to miRNA expression in parental Treg cells.

Table S2, related to Figure 1.

mRNA transcripts in in Treg-derived exosomes

A list of selected mRNAs identified in Treg cell-derived exosomes with fold change of mRNA expression relative to mRNA expression in parental Treg cells.

Table S3, related to Figure 3.

Transcriptional changes in *Dicer*$^{-/-}$ T cells following co-culture with WT Treg cells.

mRNA and miRNA expression in *Dicer*$^{-/-}$ T cells after culturing with WT Treg cells, relative to *Dicer*$^{-/-}$ T cells cultured alone.

Supplementary Experimental Procedures

***Assessment of colitis and systemic inflammation*.** Mice were monitored daily and weighed weekly. Any mice showing clinical signs of severe disease were sacrificed according to the United Kingdom Animals Scientific Procedures Act of 1986. Analysis of disease was carried out 5-weeks post cell transfer. For histopathological analysis tissues were removed, fixed in 4% paraformaldehyde in PBS, embedded in paraffin and stained with Hematoxylin and eosin (H&E), Giemsa or Alcian Blue-Periodic Acid Schiff, as indicated. Cells were recovered from various tissues as indicated and re-stimulated for cytokine analysis, as below. For Let-7d inhibitor treated Treg transfer experiments, primary Treg cells were FACS-purified from WT mice and transfected with Let-7d inhibitors with 100nM of FL-dsRNA (Thermo Scientific Dharmacon). Mock or Let-7d inhibitor transfected cells were then washed extensively with 10^5 cells transferred i.v. into recipient mice on week 2, 3 and 4.

In vitro cell culture, reagents and stimulation assays. Primary cells were isolated from naive or diseased spleen, lymph nodes or tissue, as indicated, by mechanical disruption and stained with anti-mouse CD4 (RM4-5), CD3ε (17A2), CD44 (IM7), CD25 (PC61), CD45.1 (A20), CD45RB (16A), CD19 (1D3), CD8 (53-6.7) antibodies diluted in PBS with 0.1% FCS. For Treg analysis, cells were stained with CD103 (M290), GITR (DTA-1), KLRG1 (2F1), OX40 (OX-86), CTLA-4 (eBio20A), LAG-3 (eBioC9B7W), BTLA (6F7), ICOS (7E.17G9), Granzyme-B (16G6), Foxp3 (FJK-16s). For in vitro T cell polarization experiments, FACS-purified naïve T cells ($CD4^+CD44^-$ $CD62L^{hi}CD25^-$*Ifnγ*$^{yfp-}$ or *Il17*^{Cre}*R26Re*$^{FP635-}$*Il4gfp*$^-$) were FACS purified and polarised under T_H1 (IL-12, 10ng/ml; anti-IL-4, 10μg/ml) or T_H17 (IL-6, 10ng/ml; TGFβ, 1ng/ml; anti-IFNγ, 10μg/ml, anti-IL-4, 10μg/ml) conditions, prior to FACS sorting of cytokine$^+$ cells. Unless otherwise stated, Th1 cells were FACS purified $CD4^+CD44^+$*Ifnγ*$^{yfp+}$ cells. For intracellular cytokine staining, cells were stimulated with PMA (5ng/ml) and Ionomycin (500ng/ml) for 3 hours with Brefeldin A (5μg/ml) added after 1 hour. Cells were

subsequently fixed and permeabilised with BD Cytofix/cytoperm kit (BD). In some assays, primary FACS-purified Treg cells were treated with IL-2 (high dose- 500ng/ml), IL2complex (IL-2, 5ng/ml in complex with anti-IL-2, JES6-1A2, 50μg/ml), all-trans retinoic acid (AT-RA,10nM), amphiregulin (AREG, 100ng/ml), Monensin (10μM), GW4869 (10μM) or under hypoxic conditions (1% O_2) in a hypoxia workstation chamber (Ruskin $Invivo_2$ 400, Ruskin). AlamarBlue (Invitrogen) was used to monitor cellular density / proliferation inferred from the metabolic activity, according to manufacturers guidelines. For Cox-2 inhibition, Celecoxib or DMSO, was added to T_H1-polarising cultures on day 0. IFNγ and cellular proliferation was determined on day 3.

Microarray analysis. For microarray analysis, cDNA was generated from total RNA and quality was determined using an Agilent BioAnalyzer. cDNA was hybridized to Affymetrix GeneChip mouse 430.2 arrays or mouse miRNA 3.0 arrays. Hybridization, fluidics and scanning were performed according to standard Affymetrix protocols (http://www.affymetrix.com). All cel files, representing individual samples, were normalized by using the sketch RMA method within expression console (http://www.Affymetrix.com) to produce the analysed cel files. The array data were then normalized and analysed using Agilent GeneSpring software. An ANOVA was performed to obtain multiple test corrected p-values using the false discovery rate method at the 0.1 significance level and was combined with fold change values ≥2.0. The resulting data were analysed with IPA (Ingenuity® Pathway Systems, www.ingenuity.com).

miRNA mimic and hairpin inhibitor transfection. Cells were rested in exosome-free media, washed and co-cultured with T cells, B cells or dendritic cells, as indicted, for 24-48 hours. In some experiments, cells were stained with celltrace violet (5μM, Invitrogen), cultured in separate compartments separated in a transwell system with 0.4μM pore size (Corning) or cultured with both T cells and dendritic cells for 24 hours. For confocal microscopy, transfected and non-transfected cells were stained with anti-mouse CD45.1 and analysed using a Leica SP1 confocal microscope.

Exosome isolation and analysis. Throughout these studies, complete media with exosome-depleted FCS (following 100’000g centrifugation) was used (Thery et al., 2006). Exosomes were purified by a combination of ultracentrifugation and using Exoquick solution (SBI System Bioscience) (Taylor et al., 2011), as previously reported and tested (King et al., 2012). Cell supernatant was centrifuged at 300g for 10 minutes. Cell-free supernatant was further centrifuged at 2,000g and then 10,000g to remove dead cells and cell debris. Exosomes were then purified from the resulting supernatant using Exoquick solution (Taylor et al., 2011). Exosomes recovered by this method were functionally comparable to exosomes recovered by 100’000g ultracentrifugation method (**Figure S6**). Exosomes were quantified using a CD63, CD9 and CD81 ELISA, with a standard curve of known $CD63^+$ $CD9^+$ or $CD81^+$ exosomes, respectively (SBI System Bioscience). In some experiments, exosomes were stained with anti-CD63 (NVG-2) and anti-CD9 (MZ3) and analysed using a BD LSR II flow cytometer. The size distribution of purified exosomes was also confirmed using dynamic light scatter (DLS, Malvern instruments). For in vitro assays, a titration of purified exosomes from 10^4 to 5 x 10^7 was used with 10^4 T_H cells (**Figure S6**). In all other experiments, unless indicated, 10^7 T cell-derived exosomes were used.
All FACS analysis was performed using a BD LSRII and TreeStar FlowJo and all cell sorting was performed using a BD Aria II with CellQuest software.
